# Smart Animal Welfare: A Review of Sensing Technologies, Deployment Challenges, and AI-Driven Insights

**DOI:** 10.3390/s26175387

**Published:** 2026-08-26

**Authors:** Samuel P. Mason, Ning Wang, Janeen L. Salak-Johnson

**Affiliations:** 1 Department of Biosystems and Agricultural Engineering, Oklahoma State University, Stillwater, OK 74078, USA; samumas@okstate.edu; 2 Department of Large Animal Clinical Sciences, College of Veterinary Medicine, Michigan State University, East Lansing, MI 48824, USA; john8880@msu.edu

**Keywords:** machine learning, precision livestock farming, embedded system design, welfare characteristic interpretation, physiological event detection, sensor modalities, practical deployment, dataset analysis

## Abstract

**Highlights:**

**What are the main findings?**

- Focused review of sensing, Data Acquisition, and Machine Learning methodologies for welfare-oriented Precision Livestock Farming systems.
- Critical evaluation of real-world deployment limitations in commercial livestock environments—scalability, robustness, and generalizability of sensing systems.

**What are the implications of the main findings?**

- Distinction between measurable physiological responses and broader welfare interpretation.

**Abstract:**

Precision livestock farming (PLF) integrates sensing technologies, data acquisition (DAQ) systems, and machine learning (ML) frameworks to continuously monitor individual animals and support welfare assessment through physiological and behavioral observations. Advances in infrared thermography, radar sensing, vision-based systems, acoustic monitoring, and wearable technologies have substantially expanded the ability to collect high-resolution data describing animal responses to internal and external stimuli. However, despite considerable technological progress, a persistent gap remains between sensing performance demonstrated under controlled experimental conditions and reliable deployment within commercial livestock environments. This gap is characterized by environmental variability, unrestricted animal movement, and operational constraints within commercial environments. Using a structured review methodology, this review examines sensing modalities, embedded DAQ architectures, communication strategies, ML methodologies, data privacy, farmer adoption, and an illustrative engineering workflow through the lens of welfare-relevant physiological characteristics. Emphasis placed on the distinction between direct sensor measurements and the biological processes they represent. Sensor outputs do not directly quantify welfare, stressors, or management outcomes; rather, they provide measurements of physiological and behavioral responses that require appropriate biological context for meaningful interpretation. As a result, welfare assessment does not depend solely on the ability to acquire data, but also on the ability to accurately relate those data to underlying physiological mechanisms. Within this framework, ML serves as a critical bridge between measurement and interpretation by enabling the analysis of complex, multimodal datasets. Future advancement of welfare-oriented PLF systems will require stronger alignment among sensing methodologies, physiological understanding, and practical deployment realities to generate meaningful, scalable, and biologically grounded welfare assessments.

## 1. Introduction

Precision livestock farming (PLF) refers to the continuous, automated monitoring of individual animals through sensor-based technologies to support improved management of productivity and welfare [1,2]. These systems integrate various sensing modalities, data acquisition (DAQ) infrastructures, and analytical frameworks to generate real-time or near-real-time insights into the physiological state and behavior of individual animals [3,4,5]. Recent advances in sensor technologies, wireless communication, and machine-learning-based analytics have significantly expanded the capability of PLF systems. These advances enable the collection of high-resolution physiological, behavioral, and environmental data at the individual animal level [3,6]. As these technologies mature, their integration has the potential to transform observation-based animal management into proactive, data-driven animal welfare optimization, potentially reducing disease burden, improving production efficiency, and enabling earlier intervention. Understanding the current landscape of sensing technologies, deployment challenges, and AI-driven analytical approaches is essential for unlocking the potential of fully implementing PLF in commercial settings. 

Sensing technologies have been extensively studied across numerous PLF applications, including wearable devices, non-contact sensors, and environmental monitoring systems [5,7,8,9]. Wearable sensors, such as radio-frequency identification (RFID), accelerometers, and Global Positioning System (GPS) devices, are directly attached to individual animals to monitor their movement, location, and physiological signals [2]. Non-contact modalities, such as infrared thermography, radar, machine vision, and acoustic sensors, allow observation without physical attachment. Environmental monitoring systems deliver real-time data on ambient conditions, including temperature, humidity, air quality, and hazard detection. Collectively, these technologies focus on critical indicators of animal welfare and productivity, including activity levels, thermal regulation, respiration, and feeding behavior [9,10,11].

Many of these technologies have demonstrated high accuracy in detecting specific animal behaviors and health conditions, such as lameness, thermoregulatory responses, and altered activity patterns. However, this level of success has largely been achieved under controlled or semi-structured experimental conditions. In these settings, limited movement and proximity of sensors enhance data collection and model effectiveness, while significantly reducing the operational variability found in commercial environments [2,12]. Therefore, a significant gap remains between demonstrated experimental capabilities and reliable deployment in production livestock systems [3,13,14]. Commercial livestock operations face substantially more demanding conditions. Dynamic and unpredictable factors, including variable lighting, physical obstructions, dust, extreme weather, and unrestricted animal movements, create significant challenges for sensor robustness, data integrity, and system scalability [4,15,16,17]. The practical implementation is also constrained by infrastructure and operational bottlenecks, including high power consumption, limited communication bandwidth, and intensive maintenance requirements [1,18]. Unless the cost-to-benefit ratio of these systems improves, scaling beyond small-scale or heavily subsidized operations remains economically unviable.

Another major limitation in current PLF research is the interpretation of sensor data related to animal welfare [3,19]. Many sensing approaches focus on detecting physiological or behavioral responses broadly categorized as “stress.” This often fails to adequately distinguish between transient, adaptive, or sustained responses, which have significantly different implications for welfare [12,19,20,21,22]. Stress responses can be acute or chronic and may have positive, negative, or negligible effects on welfare. This directly depends on the type, frequency, magnitude, and duration of the stressor, as well as the animal’s physiological state and capacity to recover [19]. Therefore, sensor outputs should be interpreted as indicators of underlying physiological regulation and behavioral adaptation. Thus, sensors offer insights into an animal’s response to a stimulus rather than serving as direct measurements of the welfare state itself [3,20]. Expanding the interpretation of sensor-derived data to encompass environmental factors, management variables, and production-related factors without adequate physiological justification raises considerable risks of overgeneralization and misinterpretation. Due to the highly interconnected nature of livestock production systems, measurable parameters rarely reflect isolated processes; instead, they capture overlapping biological, environmental, and management influences [12,21,23]. Attributing sensor outputs directly to specific production outcomes or management decisions may oversimplify the underlying mechanisms and lead to erroneous conclusions, potentially resulting in ineffective management decisions. For example, a decrease in feeding activity detected by a sensor might indicate pain, respiratory disease, social competition, or environmental stress. Conflating these different causes can lead to misguided interventions and delayed treatment of the true underlying conditions [24].

Animal welfare is not determined solely by the presence or absence of physiological stress responses, but by the biological significance of those responses within the context of maintaining normal function [24,25]. Physiological regulation is dynamic, with animals continuously adapting to environmental, metabolic, and behavioral demands through mechanisms collectively described as allostasis [24]. Consequently, measurable physiological characteristics should be interpreted relative to adaptive baselines rather than static thresholds. This is because identical physiological responses may represent normal adaptation in one context but compromised welfare in another [24]. This distinction provides the biological framework for interpreting sensor-derived measurements throughout this review.

Artificial intelligence (AI) and machine learning (ML) have emerged as essential analytical tools for processing and relating the extensive, multimodal datasets generated by PLF systems at fine spatial and temporal resolutions [3,13,26,27,28]. AI-driven models have been utilized for behavior classification, disease prediction, and anomaly detection and have demonstrated high performance on well-curated datasets derived from controlled studies [12,13,23,29]. These methodologies present the opportunity to advance commonly used simple threshold-based monitoring into predictive systems that can interpret and integrate large-scale, complex data streams [29,30]. However, a critical constraint on the applicability of AI in livestock settings is limited generalizability of the model—specifically, the ability of a trained model to maintain predictive performance across diverse datasets and conditions that extend beyond those represented in data sets used for model training. Models developed in highly controlled environments frequently fail to cover the model generalization required for the dynamic, heterogeneous conditions of commercial livestock operations [13,15]. The potential of AI to advance animal welfare lies in improving sensor detection and fostering a more nuanced understanding of broader animal responses [31]. By integrating data across various sensing modalities and over time, AI systems may help identify patterns associated with meaningful changes in animal conditions that would be difficult to detect using single sensor or threshold-based approaches [3,19,20,29]. Realizing this potential requires addressing the constraints inherent in both sensing technologies and analytical frameworks. However, maintaining a clear distinction between measurable physiological responses and the broader, interconnected concept of animal welfare is key to maximizing AI and ML performance.

This review examines the front-end components of precision livestock farming systems, focusing on sensing modalities, data acquisition architectures, and machine learning methodologies as they relate to welfare-relevant physiological traits. The emphasis is on assessing the practical effectiveness, deployment challenges, and physiological interpretability of these technologies in commercial livestock settings rather than under controlled experimental conditions. Sensing modalities discussed include infrared thermography, radar systems, vision-based monitoring, acoustic sensing, and wearable technologies. Embedded acquisition systems, communication architectures, and analytical frameworks such as supervised learning, deep learning, time-series analysis, and multimodal data integration are also widely discussed.

The objective of this review is to critically assess the current capabilities and limitations of PLF sensing technologies and identify critical areas requiring further development. By emphasizing physiological grounding, practical deployment considerations, and system-level interpretability, this review aims to support the development of more robust, scalable, and biologically meaningful PLF systems that generate operationally reliable data for welfare-oriented applications.

## 2. Review Methodology and Literature Selection

### 2.1. Literature Retrieval Criteria

This review was conducted as a critical narrative review to evaluate sensing technologies, data acquisition (DAQ) systems, and machine learning (ML) methodologies used for welfare-relevant monitoring in precision livestock farming (PLF). Rather than providing an exhaustive systematic review of all the available literature, the objective was to synthesize current knowledge, identify common technological and physiological limitations, and evaluate the translational challenges associated with deploying sensing systems in commercial livestock environments.

To maintain consistency and scientific rigor, the literature was selected according to criteria emphasizing relevance, methodological quality, practical implementation, and physiological interpretation. Preference was given to studies that validated sensing technologies under experimental or commercial livestock conditions or that provided comprehensive reviews of sensing modalities, embedded systems, or machine learning applications within PLF.

#### Inclusion Criteria

Studies included in this review satisfied one or more of the following criteria:

- Relevance to the review objective: Publications addressed at least one major component of welfare-oriented precision livestock farming, including sensing technologies, embedded data acquisition systems, physiological signal processing, machine learning methodologies, or welfare assessment.
- Physiological and engineering significance: Preference was given to studies that established relationships between measured physiological or behavioral characteristics and sensing methodologies, or that discussed engineering considerations affecting practical deployment.
- Publication timeframe: The review primarily emphasizes developments published from 2025 onward, while incorporating foundational literature that dispersed across many decades where necessary to provide historical context and describe the evolution of sensing technologies and physiological interpretation.
- Article type: Sources included peer-reviewed journal articles, review papers, technical reports, and graduate theses that contributed directly to sensor development, physiological validation, or system implementation. Some included sources are technical documentation from a company product package.
- Language: Only publications written in English were considered.

### 2.2. Literature Selection Process

#### 2.2.1. Database Search

Literature research was conducted primarily using Google Scholar and MDPI to obtain broad coverage of engineering, agricultural, biological, and computer science literature relevant to precision livestock farming.

#### 2.2.2. Keyword Search

Search terms were selected to encompass the major technological components of PLF as well as physiological concepts related to welfare assessment. Representative keyword combinations included:

- “Precision Livestock Farming” OR “Precision Animal Farming”
- “Animal welfare” AND sensing
- Infrared thermography AND livestock
- Radar sensing AND livestock
- Computer vision AND livestock
- Acoustic monitoring AND livestock
- Accelerometer OR IMU AND livestock
- RFID OR rumen bolus sensors
- Embedded systems OR microcontroller AND livestock monitoring
- Data acquisition OR DAQ AND livestock
- Machine learning AND livestock welfare
- Deep learning AND livestock monitoring
- Time-series analysis AND livestock
- Multimodal sensing AND animal welfare

#### 2.2.3. Search Yield

The initial literature search across the selected databases yielded approximately 1200 potentially relevant publications. Following title and abstract screening, removal of duplicate records, and assessment for relevance to the objectives of this review, approximately 185 articles underwent full-text evaluation. Final inclusion was based on technical relevance to sensing technologies, data acquisition systems, machine learning methodologies, and welfare-oriented precision livestock farming. Ultimately, 77 references were selected for inclusion, representing original research articles, review papers, and other technical publications. This selection aims to collectively provide historical context, current state-of-the-art developments, and emerging research directions. This selection process prioritized technical quality, physiological relevance, and practical applicability over exhaustive inclusion of all available literature.

#### 2.2.4. Literature Evaluation

Following the initial search, publications were evaluated for relevance to the objectives of this review. Titles and abstracts were first screened to assess relevance to welfare-oriented sensing technologies, and full-text evaluations were performed for studies considered directly applicable. Emphasis was placed on publications that reported sensor validation, physiological interpretation, embedded system implementation, or machine learning methodologies applicable to commercial livestock systems. In some cases, the literature that did not emphasize these points was still included to demonstrate the gap in understanding.

Rather than attempting to comprehensively catalog every published sensing technology, this review intentionally emphasizes studies that provide meaningful insights into the relationships among sensing methodology, physiological interpretation, practical deployment, and animal welfare assessment.

## 3. Characterization of Stressors and Welfare Relevance

The Five Domains Model provides a comprehensive framework for assessing animal welfare, structured around four physical and functional domains: nutrition, environment, health, and behavior, along with a fifth domain representing the animal’s mental state [32]. Within these physical domains, stressors are internal or external factors that disrupt an animal’s baseline biological functions. Importantly, the impact of a stressor is influenced not only by its intensity, but also by the animal’s perception, past experiences, and current physiological state [33]. These factors collectively determine the animal’s ability to cope with and recover from adverse conditions. Ultimately, animal welfare is characterized by the integrated affective experience within the mental domain, which emerges from the cumulative, interactive outcomes of the four physical domains [32]. This systemic interaction has profound implications for PLF systems. Because welfare represents an integrated mental state rather than an isolated physiological parameter, no single sensor output can serve as a direct, standalone indicator of an animal’s overall welfare status. Instead, these distinct data streams must be comprehensively integrated and interpreted through the multidimensional lens of the Five Domains Model.

While the Five Domains Model provides the conceptual framework for assessing animal welfare, interpreting physiological measurements requires an understanding of how animals maintain biological stability in response to changing internal and external conditions. Homeostasis describes the baseline maintenance of relatively stable physiological conditions through biological feedback processes [25]. However, livestock continuously encounter environmental, metabolic, behavioral, and social challenges that require physiological adjustment rather than maintenance of a static equilibrium. These adaptive regulatory processes are collectively described as allostasis, whereby multiple physiological systems actively respond to anticipated or experienced demands to preserve basal function [24]. Thus, measurable physiological characteristics such as respiration rate, heart rate, body temperature, or activity should not be interpreted relative to a single static “normal” value, but rather within an adaptive physiological baseline that reflects the individual’s current biological state and environmental context [24,25,32].

**Figure 1:**
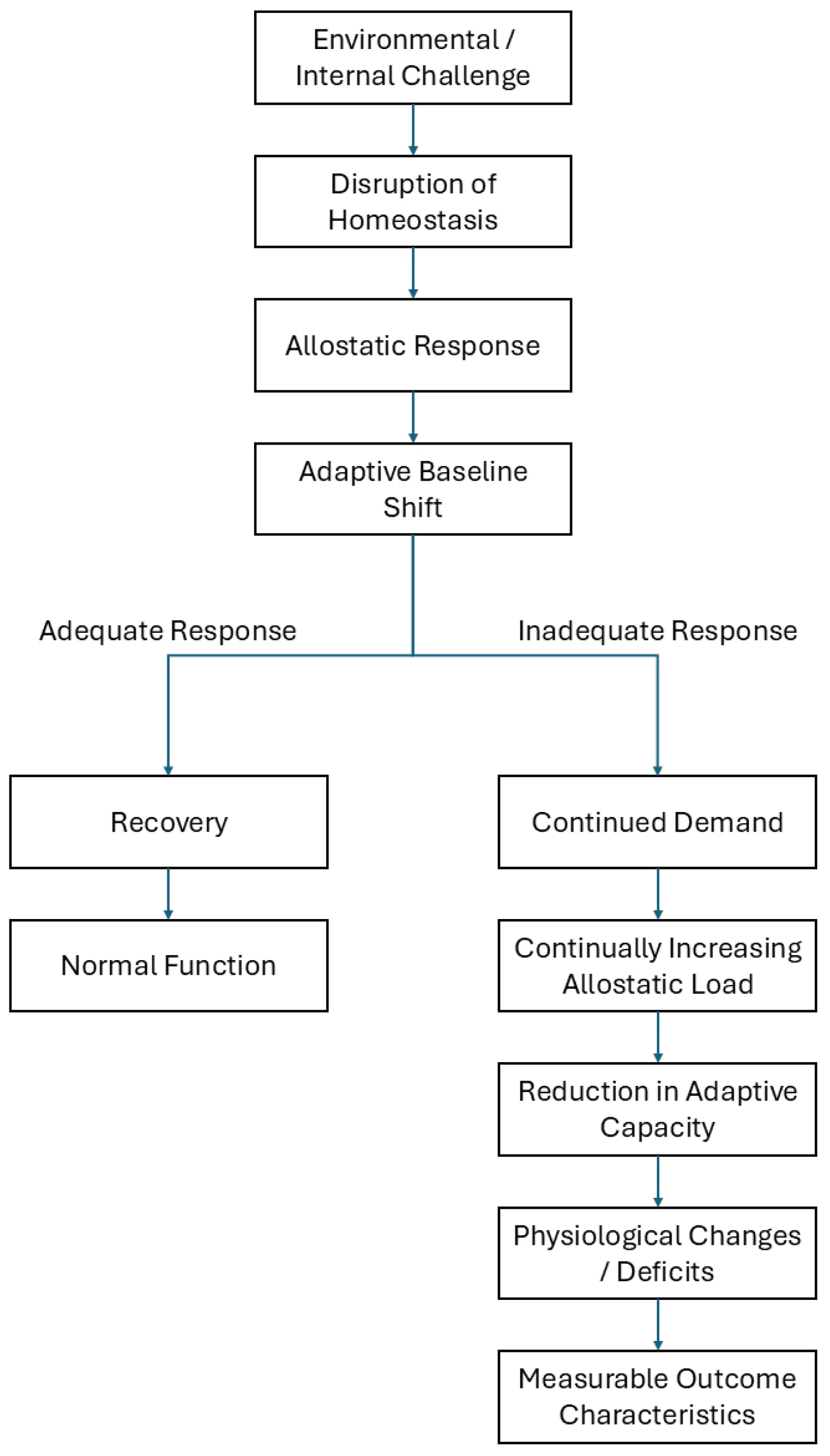
Allostatic Load Flow Diagram.

The biological cost of maintaining these adaptive responses is equally important when interpreting sensor-derived data. Short-duration physiological adjustments are often necessary components of normal adaptation and may carry little welfare significance when followed by appropriate recovery [24]. However, repeated or sustained activation of regulatory mechanisms can increase the cumulative energetic and physiological burden on the animal, a concept commonly referred to as allostatic load [24]. As this burden accumulates, the animal’s capacity to effectively respond to additional challenges may progressively diminish, increasing the likelihood of compromised health, altered behavior, and reduced welfare. These concepts are demonstrated in Figure 1. Therefore, sensor systems should not simply identify whether physiological responses occur, but should instead characterize their magnitude, duration, recovery trajectory, and broader biological context [5,34]. This distinction forms the physiological foundation for interpreting sensor outputs as indicators of welfare-relevant characteristics rather than direct measurements of welfare itself.

Further, physiological adaptation also occurs within an ecological context. Social hierarchy, resource availability, environmental heterogeneity, and interactions with conspecifics continually influence the biological demands placed upon an individual animal. Because of this, identical physiological responses may reflect fundamentally different welfare implications depending on both the individual’s internal state and the ecological conditions under which those responses occur [5].

Accordingly, an objective welfare assessment must consider not only the immediate onset of a stressor but also the duration, intensity, and cumulative effects of the biological response over time [19,34]. To bridge sensor capabilities with animal welfare assessment models, it is critical to differentiate between the external stimulus (the stressor) and the internal biological reaction of the animal (the stress response). While a stressor triggers the initial disruption, the magnitude, duration, and subsequent recovery profile of the stress response are heavily mediated by the animal’s individual physiological and psychological coping mechanisms [33]. Table 1 provides a framework illustrating the welfare relevance of measurable physiological stress responses. Rather than broadly associating all measurable physiological activation to negative welfare outcomes, this framework recognizes that stress responses span a broad spectrum, ranging from mild, transient regulation to sustained disruption of basal physiological functions. The magnitude, duration, and recovery trajectory define stressor responses, and recovery capacity is further shaped by the individual animal characteristics and physiological state at the time of exposure. Hence, it is essential for interpreting sensor-derived data within an appropriate biological context [5]. This framework is an adaptation of the Mellor Model [32] of the Five Domains suited specifically for the determination of physiological characteristics’ ability to be sensed.

**Figure 2:**
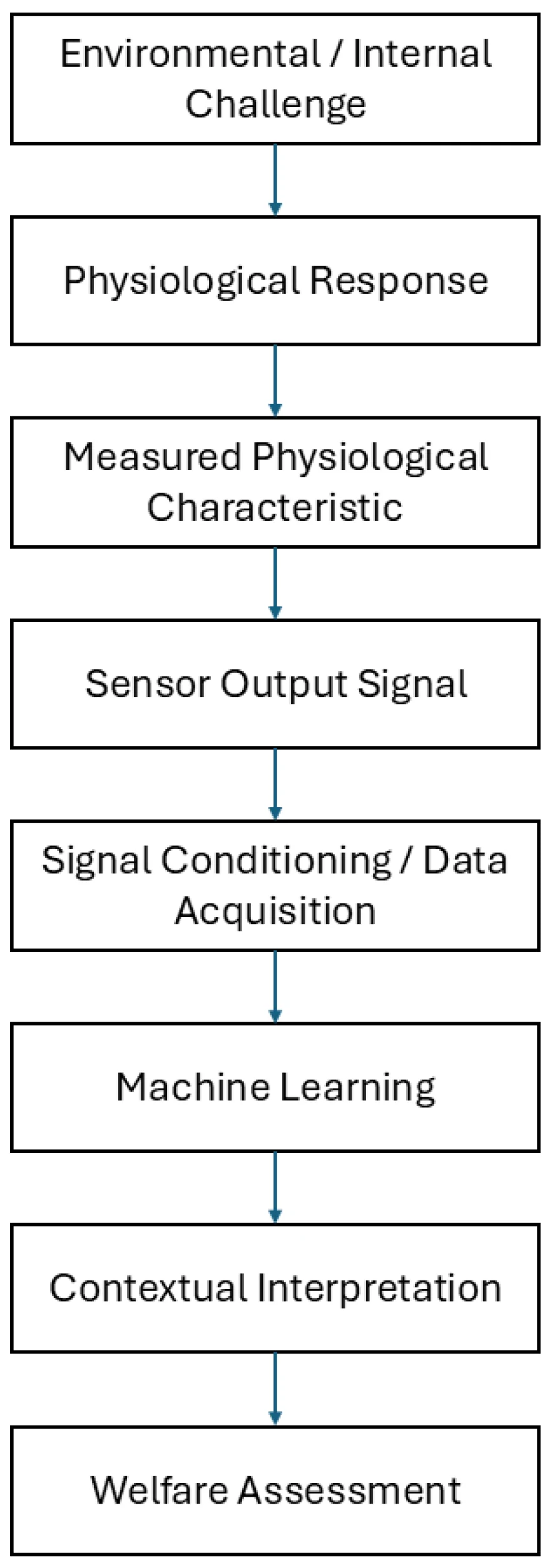
Simplified Physiology-Sensor-Assessment Framework.

To bridge these concepts, Figure 2 introduces a simplified primary flow of how allostatic load and physiological concepts are combined with sensing and then interpreted into a relevant welfare assessment.

## 4. Sensor Modalities

The sensing modalities discussed throughout this section acquire information spanning both physiological indicators and behavioral proxies. These each contribute distinct and complementary information for welfare assessment. Physiological indicators, such as respiration rate, heart rate, and body temperature, are measurable biological processes. These processes generally provide a shorter inferential pathway to underlying physiological regulation. In contrast, behavioral proxies, including feeding activity, locomotion, posture, and social interactions, require additional interpretation. This is because they are influenced by environmental conditions, management practices, and ecological context. Importantly, neither measurement type directly quantifies animal welfare. Rather, both represent complementary sources of information whose biological significance depends on appropriate physiological interpretation and contextual integration. In this review, the terms physiological indicators and behavioral proxies are utilized with recognition that both require distinct inferential pathway lengths based on the deployment context.

**Figure 3:**
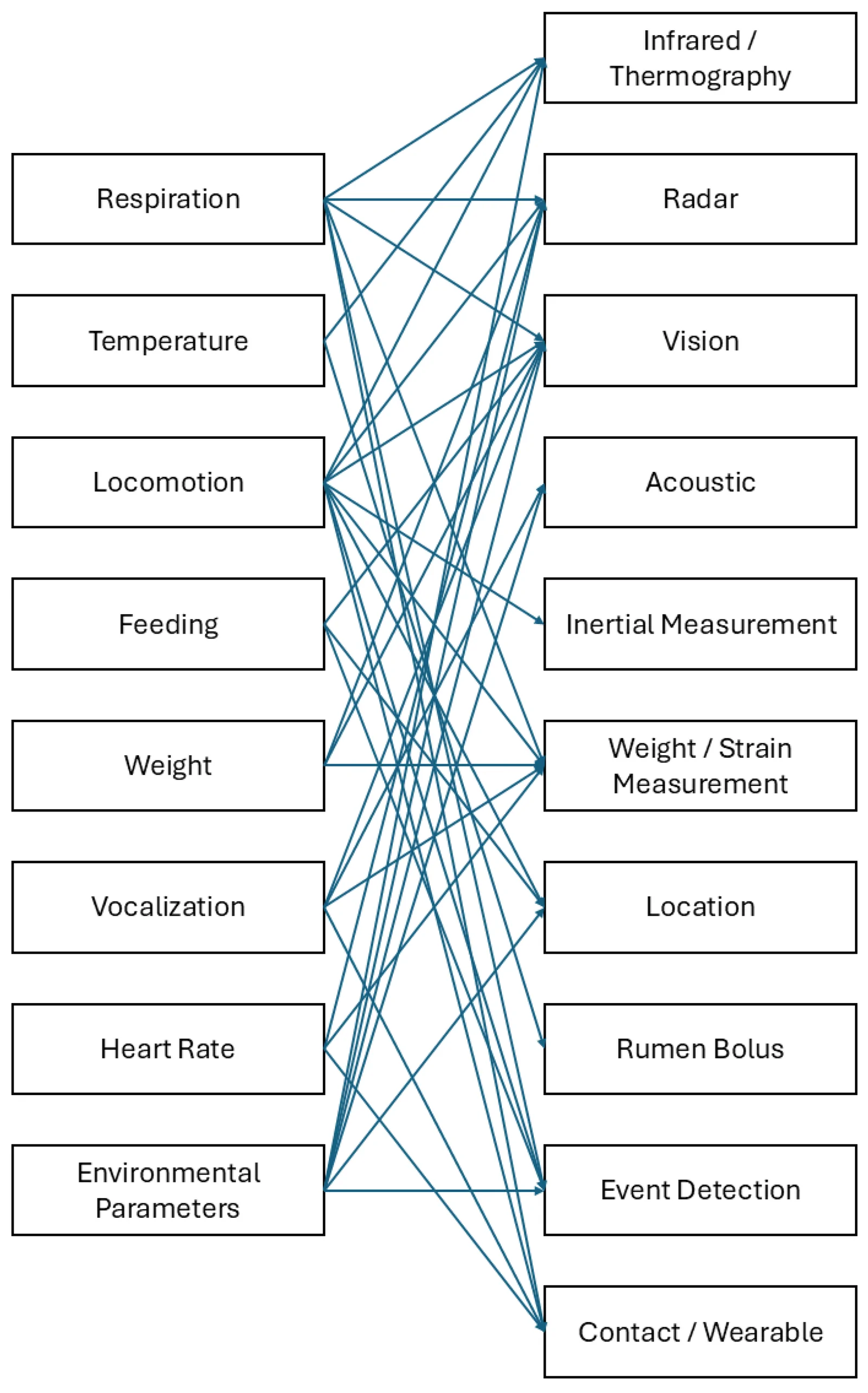
Web Chart Connecting Parameters (**left**) to their Sensors (**right**).

Table 2 summarizes the primary sensing modalities used in PLF applications with their key advantages and deployment limitations. Many sensing technologies are first developed and tested in human medicine or other fields before being applied to livestock. Therefore, this review includes relevant studies from both livestock and non-livestock applications. Studies outside of livestock are used to describe the sensing principles, capabilities, and development, but they are not considered as direct evidence that the technology has been validated for livestock. This allows us to consider if a sensing technology has potential to be transferable to livestock applications. Figure 3 demonstrates the interconnectedness of sensors and sensible parameters in PLF. The sections that follow discuss each modality in depth, with particular attention to practical effectiveness under commercial conditions and the physiological interpretability of the data each technology generates. Regardless of sensing modality, practical implementation requires consideration of the interaction between the sensing system and the animal itself. Things like attachment methods, enclosure design, and deployment strategies may influence both measurement quality and the physiological responses being monitored [29].

### 4.1. Infrared Temperature Sensor and Thermal Imaging System

Infrared Temperature Sensor (IRT) and Thermal Imaging System (Thermography) have proven to be effective for non-invasive measurements of biological temperatures, particularly for assessing surface temperature distributions on animal bodies [10,43]. Surface temperature measurements can correlate with core physiological parameters, such as core body temperature. This is done by targeting localized regions with high superficial microvascular density, including the ocular and nasal areas, where cutaneous blood flow most reliably reflects internal thermal state [10,35,43,44]. These correlations have been applied to assess thermoregulatory responses in livestock furthering the use case for IRT and Thermography as proxy measurement tools for thermally relevant welfare parameters [5,10,44].

Despite showing great promise, IRT systems require precise and structured measurement conditions that can greatly limit the efficacy of practical deployment. Things like accuracy are influenced by factors including distance, angle, emissivity assumptions, and stability of the target region [10]. Many IRT systems attain consistent measurements by leveraging predictable animal positioning. For example, during feeding at a designated feeding station or within chute-based handling systems, movement is limited and motion artifacts are minimized [45]. While IRT systems can yield high-quality data, their applicability in settings where animal movement is unrestricted is constrained.

Wider-field thermal imaging systems enable simultaneous evaluation of multiple animals over greater distances and across larger areas [43,45]. However, this expanded coverage reduces precision, as environmental factors, including ambient temperature, solar radiation, airflow, occlusion, and surface contaminants, introduce measurement variability that is more difficult to control at scale [10]. Its accuracy is further diminished by individual animal variations in posture and coat characteristics, including color and density, which can influence surface emissivity and the interpretation of thermal signals. Despite these limitations, the capability to continuously capture spatially distributed temperature data across individuals or groups offers a scalable pathway for integration with machine learning frameworks [46]. Large-scale thermal datasets may support pattern recognition approaches to identify trends associated with changes in physiological state, including the onset and progression of thermoregulatory stress. However, the reliability of such systems depends on robust preprocessing, environmental normalization, and careful interpretation of temperature as a proxy for internal physiological state rather than a direct measurement [10,43].

In addition to environmental normalization, IRT and Thermographic systems can quantify group-level interactions through heat density mapping and thermal distribution. Thermal relationships may provide environmental/ecological context that is valuable to complement physiological measurements and improve interpretation of welfare-related responses [23].

### 4.2. Radar Sensors

Recently, microwave and radar-based sensing systems have shown increasing promise for non-contact monitoring of physiological parameters, including respiration rate and heart rate, by analyzing subtle movements of the body surface and chest wall displacement [37,47]. These systems operate by transmitting radiofrequency signals and analyzing phase or frequency shifts in the reflected signal, enabling detection of periodic biological motion even under low-visibility conditions [37,47]. Unlike optical systems, radar-based approaches function effectively in environments with dust, fog, or partial occlusion, making them particularly well suited for livestock monitoring applications [37].

Frequency-Modulated Continuous-Wave (FMCW) and Doppler radar systems offer high accuracy in controlled environments, particularly when animals are stationary or isolated, which facilitates effective signal separation and minimizes interference [37]. Ultra-wideband (UWB) radar systems further enhance this capability by enabling spatial localization in addition to physiological monitoring, supporting multi-parameter sensing within a defined area [38]. However, deploying radar sensing in dynamic environments presents considerable challenges in isolating signals from individual animals within a shared sensing area [38,47].

The resolution of radar-based systems is inherently linked to wavelength and system configuration, which can limit precision when extracting fine-scale physiological signals under unconstrained conditions [37]. While these systems offer clear advantages in robustness to environmental interference, practical deployment in livestock settings remains constrained by signal-processing complexity, hardware costs, and challenges in scaling to large populations [12,37,38]. Despite these limitations, the capacity of radar systems to generate continuous, non-contact physiological data streams presents a meaningful opportunity for integration with machine learning frameworks, particularly in applications where optical sensing is unsuitable. Recent advances in 4D imaging radar operating at millimeter-wave frequencies (77 GHz to 81 GHz) have enabled the generation of high-density point clouds by providing critical elevation data alongside standard range, azimuth, and Doppler velocity metrics [48]. When integrated with deep-learning-based perception stacks, these systems can dynamically classify the structural characteristics and boundaries of an object, maintaining high fidelity even in conditions of zero visibility such as during heavy dust, torrential rain, or thick fog [49,50]. As a result, 4D imaging radar represents a cost-effective, high-performance, non-contact sensing option with significant potential for livestock monitoring, capable of overcoming the severe environmental occlusions and lighting dynamics that traditionally compromise computer vision systems in commercial barns and pastures [51,52].

Like the capabilities of standard camera systems and thermographic systems, radar sensors can both function on an individual level or generate data for an entire group of animals. True identification of individuals proves very difficult for radar; however, the ability to identify clustering/grouping behavior may still provide valuable ecological context. This context can directly complement physiological measurements and improve interpretation of welfare-related responses [5].

### 4.3. Vision-Based Sensors

Vision-based sensing systems, including RGB and RGB-D depth cameras, have been widely used in PLF to monitor animal behavior, such as locomotor patterns and postural changes [39,53]. These systems enable non-contact, high-resolution data acquisition and are particularly effective for detecting indicators such as lameness, feeding behavior, and social interactions through image and video analysis [53]. The use of depth imaging further enhances this capability by providing three-dimensional spatial data, thereby supporting a more comprehensive evaluation of body condition, gait, and space utilization [39]. Computer vision approaches have demonstrated significant efficacy in classification and tracking tasks, particularly within intensive confinement systems, such as feedlots, swine barns, and poultry houses, where fixed infrastructure and predictable spatial layouts facilitate consistent sensor placement and controlled fields of view [54,55].

Despite these advantages, the effectiveness of vision-based systems varies considerably depending on the production environment. In intensive confinement settings, primary challenges arise from high animal density, individual occlusion (where one animal’s body blocks the camera’s view of another), and the visual complexity introduced by overlapping animals in shared space, which reduce the accuracy and reliability of individual identification and tracking [53,55]. With poultry in particular, the large number of individual birds in a confined space creates unique circumstances and challenges that can be examined with various group monitoring techniques and still produce meaningful data [31,56]. In extensive production systems, where animals range freely across open pasture or rangeland, a distinct set of challenges predominates variable and uncontrolled lighting/weather conditions, airborne particulates such as dust that degrade image quality, and unpredictable animal-to-camera distances [57]. Together, these environmental factors substantially limit the spatial resolution and consistency of data collected in extensive settings, making robust individual tracking considerably more difficult than in intensive or confined experimental environments [53].

When combined with depth camera systems, multi-camera arrays capable of producing three-dimensional reconstructions are showing promise for accurately measuring animal morphometrics. Common morphometrics to measure include body length, width, volume, and calculated weight, in real time [40]. Open-source image processing and point-cloud processing frameworks can generate such three-dimensional models from camera arrays and depth data [31,56]. However, reconstruction accuracy degrades with increasing animal density and occlusion within the detection zone, limiting their current applicability in high-density production settings [40].

Beyond individual behavioral classification, highly advanced, modern vision systems have the potential to quantify group-level interactions including spacing, competition, social cohesion, and resource access. These ecological relationships may provide valuable contextual information that complement physiological measurements and improve interpretation of welfare-related responses.

Despite these constraints, vision-based systems remain a foundational component in PLF applications due to their reasonable cost, scalability, and capacity to generate rich, continuous behavioral data streams. When integrated with machine learning approaches, the vision-based systems present considerable opportunities for automated, welfare-relevant behavioral monitoring [56]. Realizing this potential in practice, however, requires rigorous preprocessing pipelines, systematic sensor calibration, advanced data analytics, and ongoing adaptation to the dynamic and variable conditions characteristic of commercial farm environments [54].

### 4.4. Acoustic Sensors

Acoustic sensors utilize microphones and audio processing techniques to monitor vocalizations, respiration, and other sound-based indicators of animal behavior and physiological state [14,58,59]. These sensors offer a passive, non-contact method for continuous data collection and have been applied to identify various conditions. Respiratory disease, stress-related vocalizations, and feeding behavior have all been identifiable in some capacity by capturing and analyzing characteristic sound patterns [14,58]. In structured environments, the combination of acoustic analysis and machine learning has demonstrated the ability to classify vocalizations and detect abnormal respiratory sounds with varying degrees of accuracy [59]. However, the effectiveness of acoustic sensing in commercial livestock settings, both intensive and extensive, is significantly affected by ambient background noise, overlapping vocalizations from multiple animals, and variability in surrounding acoustics. All these factors reduce signal clarity and complicate automated classification [58].

Sound propagation is further affected by barn configuration and equipment noise, which complicates consistent signal acquisition and interpretation [14]. Isolating the vocalizations of individual animals within a multi-animal barn is a critical technological challenge. Without reliable individual-level measurements, acoustic data reflects the collective sound features of the group animals rather than the welfare state of a single animal [28]. Specifically with poultry applications, group level acoustics can indicate a flock stressor that can subsequently be identified on an individual level by other mechanisms [14]. This lowers the precision that PLF systems require. 

Despite these challenges, acoustic systems offer a cost-effective and scalable sensing option that can complement other modalities by providing continuous monitoring of auditory welfare indicators. When integrated with machine learning frameworks, acoustic data streams may facilitate early detection of welfare-relevant physiological changes; however, effective noise filtering, signal source separation, and contextual interpretation are essential for reliable deployment in commercial settings [28,59].

### 4.5. Animal Wearable Sensors

Animal identification is a foundational requirement for any PLF system, as it enables the attribution of sensor-derived data to individual animals over time. Radio-frequency identification (RFID) systems, e.g., RFID ear tags, are widely used in livestock production for individual animal identification and the automated recording of discrete events, e.g., feeding, watering, and passage through fixed detection points [42,60,61]. These systems generate time-stamped records of individual animal interactions with specific resources or locations within a production environment. RFID-based monitoring is particularly effective in operationally structured environments where animals routinely pass through or engage with defined sensing points, yielding reliable event-level records of animal presence and activity [42]. However, RFID systems carry several practical limitations. Their reliance on fixed infrastructure and event-driven data collection produces fragmented records relative to continuous sensing approaches, and they do not yield direct physiological or behavioral measurements. Integration with complementary sensing modalities is therefore necessary to capture the broader range of parameters relevant to welfare assessment. Physical tag retention also poses a persistent operational challenge, as ear tags are susceptible to loss due to animal-to-animal transfer and theft, compromising individual-level data continuity. Despite these limitations, RFID remains a cost-effective and scalable foundation for animal identification and data attribution within larger PLF systems [61].

Rumen bolus sensors were initially designed as another animal identification sensor. With addition of other sensors, they provide a means of ID-tagged, continuous measurements of core body temperature, rumen pH, and rumen motility from within the gastrointestinal tract [62]. These sensors offer a distinct advantage in capturing internal physiological data that are otherwise difficult to obtain non-invasively. This supports early detection of conditions such as metabolic disorders, heat stress, and digestive disturbances [62]. However, bolus sensors provide limited insight into behavior or external environmental interactions. When used in conjunction with external sensing modalities, these boluses enable a more complete characterization of individual animal condition across both internal physiological state and external behavioral or environmental impacts [5].

Accelerometers and inertial measurement units (IMUs) are among the most widely adopted wearable sensors in precision livestock farming, enabling continuous monitoring of animal activity, posture, and movement patterns [8,61,63,64]. These devices are particularly effective for detecting behavioral changes associated with lameness, estrus, and general locomotion through multi-axis acceleration data [8,12,64]. In controlled, smaller, intensive environments, accelerometer-based sensors have demonstrated strong classification performance when paired with machine learning algorithms for pattern recognition [63]. However, data interpretation is highly dependent on sensor placement, attachment stability, and algorithm design, with additional variability introduced by differences in animal morphology and individual movement patterns [8]. Furthermore, while accelerometers provide high-temporal-resolution kinematic data, they offer no direct welfare-related physiological measurements. Abnormal animal behaviors, such as pain, fever, or metabolic stress, may be derived from the continuous acceleration data. Despite these limitations, their durability, relatively low cost, and compatibility with large-scale deployment make accelerometers and IMUs a foundational component of wearable sensing systems, particularly when integrated into multimodal data pipelines [61].

Global Positioning System (GPS) and Global Navigation Satellite System (GNSS)-based sensors enable spatial tracking of individual livestock, offering insight into movement patterns, grazing behavior, and habitat utilization across large areas [7,65]. These systems are particularly valuable in extensive production systems where continuous visual monitoring of individual animals is impractical, allowing assessment of activity distribution and environmental interactions at both the individual and herd levels [7]. When integrated with accelerometer data, GPS and GNSS systems can improve behavioral classification by providing spatial context to movement patterns [65]. However, GPS-based sensing carries notable limitations. Positional accuracy is diminished in obstructed environments such as indoor housing facilities or areas with dense canopy cover. Other operational constraints like data transmission and battery longevity further limit continuous deployment [7]. Importantly, spatial data alone does not reflect physiological state. It provides contextual information about where and how animals move but it requires integration with physiologically informative sensing modalities to enable meaningful welfare interpretation.

Wearable-type sensors typically produce data streams at the individual level that do not provide insight at the group level. However, integrating synchronous streams from multiple individuals can seamlessly produce grouped data and meaningful environmental and ecological context. This group context can greatly enhance the context for individual physiological measurements.

### 4.6. Common Sensor Study Caveats, Limitations, and Shortcomings

**Figure 4:**
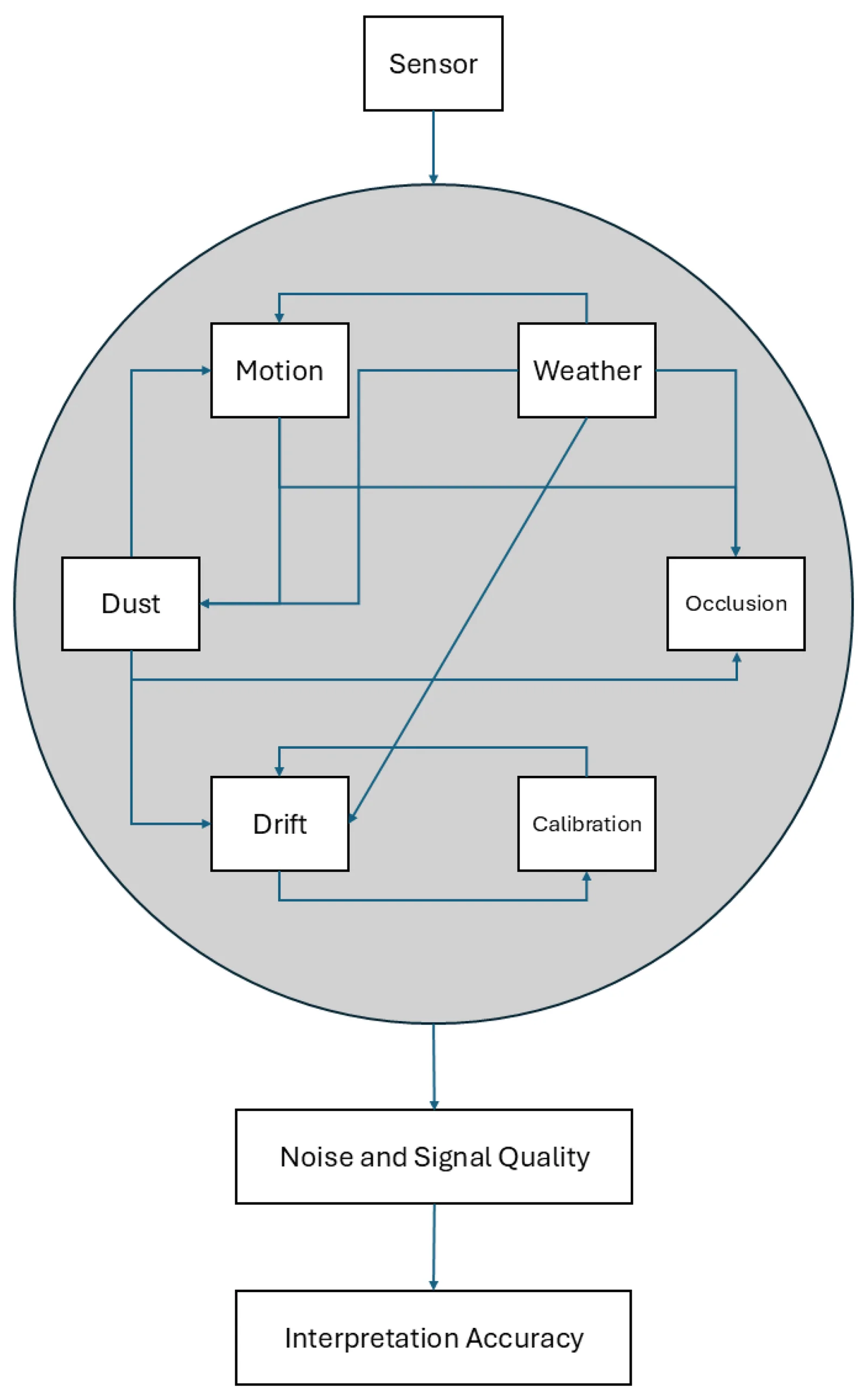
Sensor Error Visualization.

The performance of sensing systems reported throughout the PLF literature should be interpreted within the context of the experimental conditions under which they were developed and validated [66]. Reported accuracy, detection rates, and classification performance are frequently influenced by study design, including animal population, environmental conditions, sensing modality, deployment duration, validation methodology, and statistical treatment. These factors do not necessarily diminish the value of individual studies, but they do define the scope within which the reported conclusions remain scientifically valid. Recognizing these common caveats is therefore essential when comparing sensing technologies, evaluating their readiness for commercial deployment, and identifying research gaps that remain before robust, scalable welfare assessment systems can be achieved [34]. Table 3 summarizes common limitations encountered across sensing studies, while Figure 4 illustrates how these sources of uncertainty propagate through the sensing pipeline, ultimately influencing physiological interpretation and welfare assessment.

## 5. Data Acquisition (DAQ) Modalities

**Figure 5:**
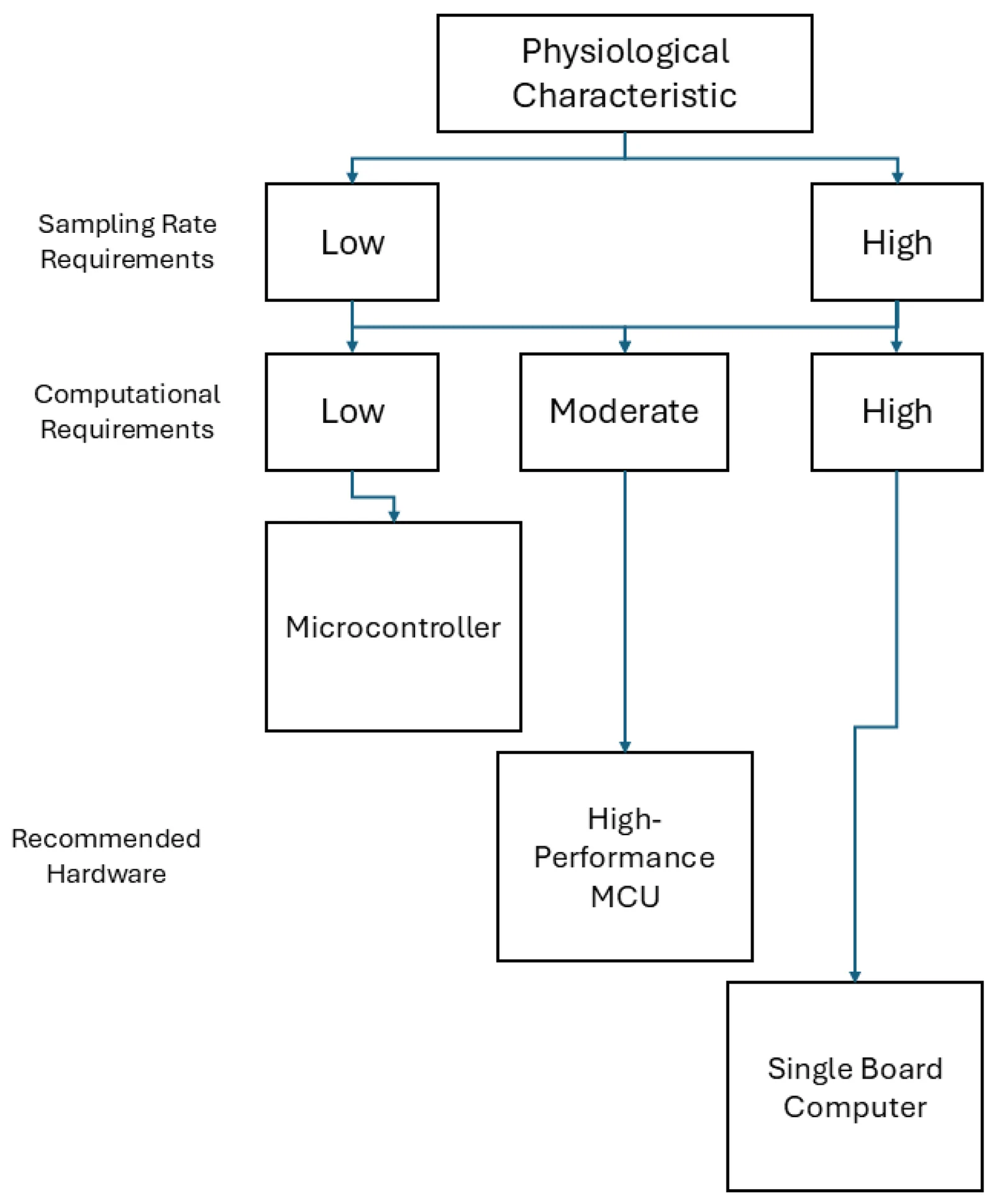
Selection Workflow and Criteria for DAQ use in PLF.

**Figure 6:**
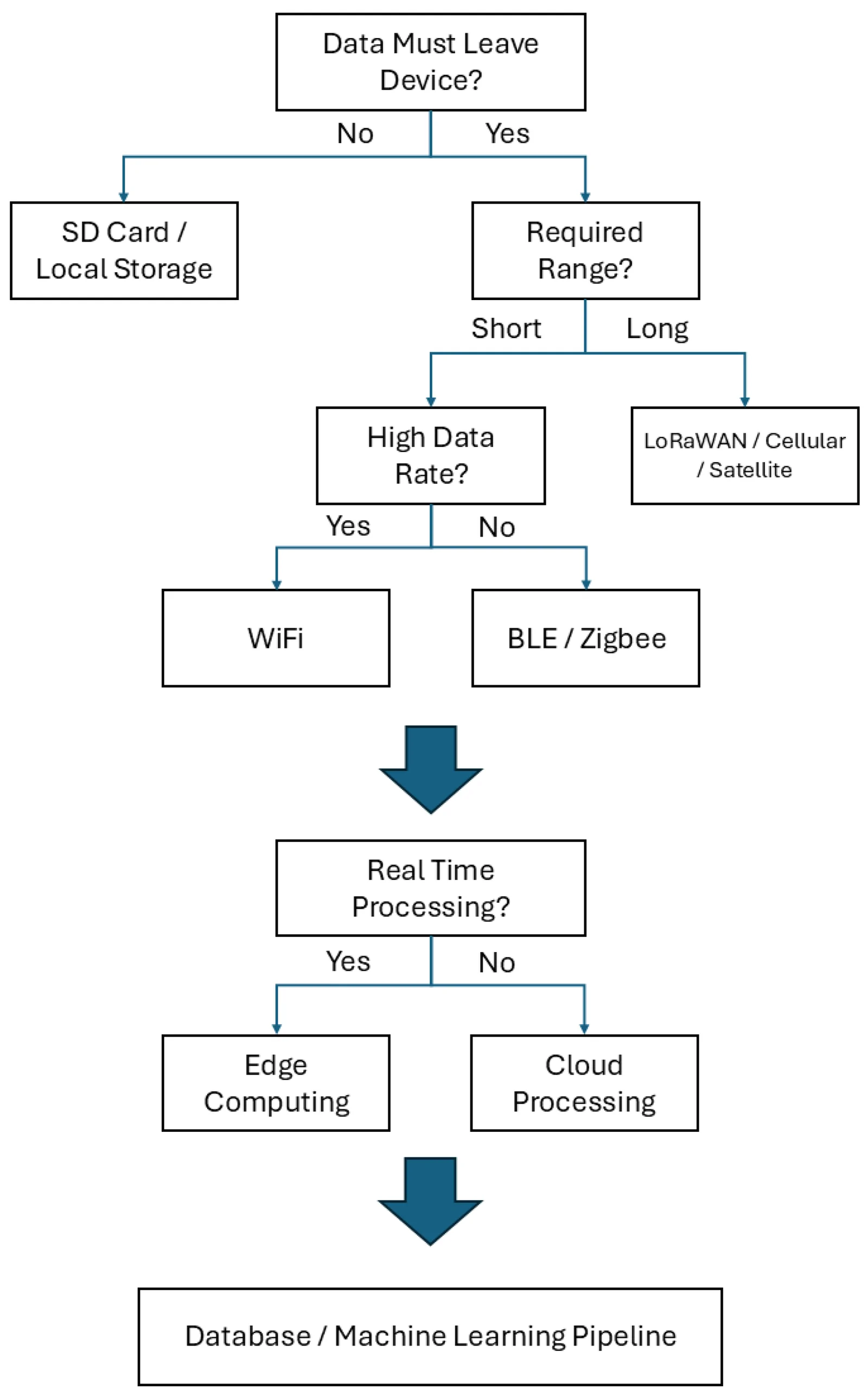
Selection Workflow and Criteria for Data Communications use in PLF.

Table 4 and Table 5 summarize the data acquisition systems and data communication strategies used in PLF applications with their key advantages and deployment limitations. Following the summary tables, Figure 5 and Figure 6 visualize selection workflow and criteria, summarizing their use in PLF. The sections that follow discuss each modality in depth, with particular attention to practical effectiveness under commercial conditions and the physiological interpretability of the data each technology generates.

### 5.1. Embedded Data Acquisition Systems

Embedded data acquisition systems serve as the foundational interface between sensing hardware and higher-level analytical frameworks in PLF systems. These platforms are responsible for sensor interfacing, signal acquisition, analog-to-digital conversion (ADC), preprocessing, timestamping, and temporary data storage prior to transmission or further analysis [6,15]. Common embedded platforms include low-power microcontrollers, microprocessors, and single-board computers (SBCs) such as the Raspberry Pi (https://www.raspberrypi.org/, accessed on 1 December 2025) and NVIDIA Jetson systems (https://www.nvidia.com/en-us/industries/robotics/, accessed on 1 December 2025), which vary substantially in computational capability, power consumption, and suitability for deployment.

Microcontroller-based systems are widely used in wearable and distributed sensing applications due to their low power requirements and compact form factors. These attributes support continuous data acquisition from low-bandwidth sensors such as accelerometers, RFID readers, and environmental sensors [16]. However, these systems are constrained by limited onboard memory, processing capacity, and sampling rates. Constraints like these greatly restrict the ability to support computationally intensive preprocessing or high-bandwidth modalities such as thermal imaging or video analysis [21].

More capable, embedded platforms like SBCs and dedicated edge-processing devices, enable localized execution of machine learning inference, image preprocessing, and multimodal data synchronization directly at the acquisition node. This improves real-time analytical capability and reduces upstream communication burden [6,31,67]. However, these advantages come at the cost of increased power consumption, thermal management requirements, and reduced environmental durability, each of which presents practical constraints on long-term deployment in commercial livestock environments.

### 5.2. Mobile and Distributed Logging Systems

Mobile and distributed logging systems enable decentralized acquisition of physiological and behavioral data across livestock environments through wearable devices, standalone dataloggers, and distributed edge nodes [18,41]. These systems are particularly valuable in extensive or semi-structured production environments where centralized acquisition infrastructure is impractical or where animal mobility necessitates localized data capture [41].

Wearable acquisition systems commonly integrate onboard sensing, temporary storage, and wireless transmission into compact, battery-powered devices attached directly to the animal [62]. This approach enables continuous monitoring of activity, location, and physiological proxies while minimizing dependence on fixed infrastructure. However, long-term deployment reliability is substantially constrained by device size, battery longevity, attachment stability, and exposure to environmental conditions.

Standalone dataloggers offer an alternative for localized data acquisition where continuous wireless communication is not feasible, enabling high-frequency recording while reducing transmission requirements [15]. This approach introduces trade-offs, however, including delays in data availability and increased logistical complexity associated with manual retrieval, synchronization, and device maintenance. More broadly distributed acquisition architectures present persistent challenges related to time synchronization across nodes, intermittent communication, and the integration of heterogeneous sensors within large-scale deployment [41,68].

### 5.3. Sampling and Signal Conditioning

Sampling strategy and signal conditioning fundamentally influence the quality, interpretability, and analytical utility of physiological and behavioral data collected in precision livestock systems [15,21]. Sensor outputs are frequently affected by environmental noise, motion artifacts, electromagnetic interference, and sensor drift, necessitating preprocessing techniques such as filtering, amplification, normalization, synchronization, and calibration prior to downstream analysis. Environmental conditions further complicate data acquisition by introducing variability across sensing modalities. Factors including temperature fluctuations, humidity, dust, lighting variability, and unrestricted animal movement can significantly degrade signal quality and consistency in real-world livestock environments [9,10].

Sampling frequency directly impacts the ability to capture physiologically meaningful temporal dynamics. High-frequency acquisition enables detailed characterization of transient events such as gait abnormalities, respiration patterns, or rapid behavioral changes, but substantially increases storage requirements, power consumption, computational overhead, and communication burden. These requirements can potentially limit long-term scalability and deployment efficiency [15]. On the other hand, lower sampling rates reduce system demands but may fail to capture short-duration physiological responses or subtle behavioral transitions relevant to welfare assessment. As a result, adaptive sampling strategies are increasingly important for balancing temporal resolution with practical system limitations [69].

Analog-to-digital conversion (ADC) resolution also affects the sensitivity and precision of acquired signals, particularly in sensing modalities that involve subtle thermal, acoustic, or physiological fluctuations [69]. However, increasing acquisition resolution and sampling frequency often produces diminishing returns when environmental variability and biological noise exceed the effective sensitivity of the sensing system. Because of this, effective DAQ design requires balancing signal fidelity with practical deployment constraints including power availability, storage capacity, communication capability, environmental durability, and long-term operational reliability.

### 5.4. Data Transmission and System Architecture

Data acquisition in precision livestock farming systems relies on both wired and wireless infrastructures to collect and transmit sensor data from distributed sources [15,18,31]. Wired systems offer high reliability, low latency, and consistent data throughput, making them suitable for fixed installations such as milking parlors or controlled feeding stations [15,67]. However, they are inflexible, and installation costs are high, limiting scalability, particularly in dynamic or extensive livestock environments.

Wireless systems, including wireless sensor networks (WSNs), offer greater flexibility and scalability by enabling distributed sensing across large and heterogeneous environments [16,18]. These systems support mobile and wearable sensors but introduce challenges related to signal reliability, interference, power consumption, and network stability [16]. As a result, the choice between wired and wireless DAQ systems often reflects a trade-off between reliability and scalability, particularly in real-world deployment conditions.

#### 5.4.1. Data Communication

Communication range is a critical factor in DAQ system design, influencing both data transmission reliability and infrastructure requirements [6]. Short-range communication protocols, such as Bluetooth and Wi-Fi, are commonly used for high-throughput data transfer in localized environments, such as barns or feeding areas [6]. These systems offer low latency and high data rates but are limited in coverage and often require dense infrastructure deployment.

In contrast, long-range communication technologies, including LoRaWAN and cellular networks, enable data transmission over large distances with reduced infrastructure requirements, making them suitable for extensive grazing systems [18,68]. However, these systems typically operate at lower data rates and may introduce latency, limiting their suitability for high-frequency or real-time monitoring applications. The choice of communication modality therefore depends on the balance between data resolution requirements and environmental scale [69].

#### 5.4.2. Edge vs. Cloud Architectures 

The architecture of data processing in PLF systems is commonly divided between edge computing and cloud-based approaches [6,13,31]. Edge computing involves processing data locally at or near the sensor source, reducing latency and bandwidth requirements while enabling real-time decision-making [6]. This approach is particularly beneficial in environments with limited connectivity or where immediate response is required [31].

Cloud-based architectures, in contrast, centralize data storage and processing, enabling large-scale data integration, long-term analysis, and more computationally intensive machine learning applications [13]. However, reliance on cloud infrastructure introduces challenges related to connectivity, data transfer costs, and system robustness in remote or infrastructure-limited environments. Hybrid approaches that combine edge and cloud processing are increasingly explored to balance these trade-offs [67].

## 6. Data Analysis and Machine Learning

Analytical approaches in PLF span a broad spectrum, from traditional statistical methods applied to small, structured datasets to advanced machine learning frameworks for processing large-scale, continuous sensor streams. Machine learning (ML) has become an increasingly integral component of precision livestock farming systems, enabling the extraction of meaningful patterns from the high-dimensional, multimodal datasets generated by modern sensing technologies [3,54]. The selection of an appropriate analytical approach is largely dictated by data volume, temporal structure, physiological context, and the intended interpretation of resulting outputs [41].

### 6.1. Small Dataset Analysis and Signal Processing

Small datasets remain common throughout livestock sensing research, particularly during sensor validation, proof-of-concept studies, and physiological characterization experiments. In these applications, traditional statistical and signal processing techniques are often more appropriate than complex machine learning approaches due to limited sample sizes and highly structured experimental conditions [34].

Statistical analyses such as correlation analysis, regression modeling, analysis of variance (ANOVA), and hypothesis testing are frequently used to establish relationships between sensor outputs and physiological reference measurements. These methods provide interpretable results and are valuable for determining sensor accuracy, repeatability, and biological relevance.

**Figure 7:**
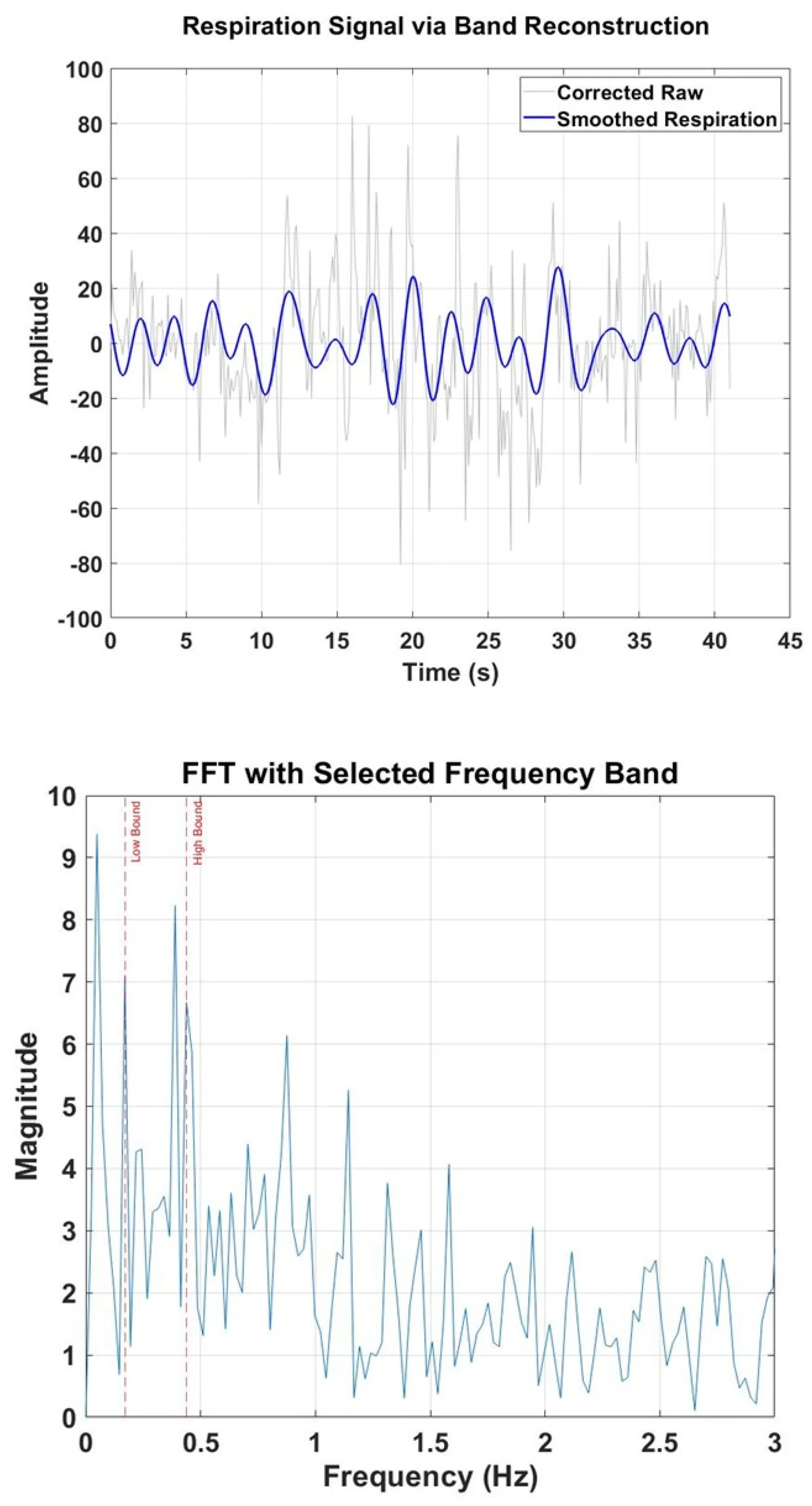
Stacked FFT Example. Raw, Linearized Data with Smoothened Inverse FFT (**top**), and FFT Result with Dominant Frequency Reconstruction Bounds (**bottom**).

Signal processing techniques additionally play an important role in extracting physiologically meaningful information from raw sensor data. Methods such as filtering, frequency-domain analysis, and Fast Fourier Transform (FFT) analysis are commonly used to characterize periodic biological processes, including respiration, locomotion, and cardiovascular activity. For example, continuous respiration monitoring systems frequently employ FFT analysis to identify dominant respiratory frequencies from short-duration sensor recordings. These approaches are computationally efficient, highly interpretable, and often serve as the foundation for more advanced analytical frameworks. An example of this is Figure 7, adapted from methods used by Mason [66].

### 6.2. Large Dataset Analysis and Event Detection

As sensing systems mature and deployment durations increase, livestock monitoring platforms generate substantial volumes of continuous physiological, behavioral, and environmental data [31,67]. Within these datasets, analytical objectives shift from simple measurement validation to identifying patterns, trends, anomalies, and state transitions that may indicate meaningful changes in an individual animal’s condition.

Large-scale datasets are particularly valuable for detecting infrequent or temporally distributed events that may not be observable within the timeframes of traditional experimental studies. For example, gradual behavioral changes preceding disease onset, prolonged thermoregulatory responses, alterations in feeding patterns, and cumulative responses to recurring environmental stressors [31]. Analytical frameworks capable of detecting and contextualizing such events serve as a critical bridge between raw sensor outputs and biologically meaningful interpretation, provided that the physiological relevance of detected patterns is explicitly defined rather than assumed from statistical association alone [17].

### 6.3. Machine Learning Applications for Large Datasets

Machine learning provides the primary analytical framework for extracting information from large-scale livestock datasets. Supervised learning methods remain widely used for classification and regression tasks where labeled datasets are available. Deep learning architectures enable automated feature extraction from complex inputs such as images, video streams, radar signals, and continuous physiological measurements [54,70].

Most livestock sensing applications are inherently temporal, since physiological responses, behavioral adaptations, and welfare-relevant characteristics evolve, rather than existing as isolated observations. Time-series modeling therefore forms the foundation of many ML applications within PLF systems, enabling the identification of trends, state transitions, recurring patterns, and anomalous events that would not be detectable from individual measurements alone [15,21].

Increasingly, ML models are incorporating multimodal data streams derived from multiple sensing technologies. Integrating thermal, visual, acoustic, wearable, environmental, and physiological data has the potential to improve analytical robustness and contextual understanding while reducing dependence on any single sensing modality [3,17]. However, multimodal integration introduces compounding challenges related to data synchronization, variable data quality, computational demand, and error propagation across modalities, all of which can degrade overall system reliability if not explicitly addressed in model design and validation.

### 6.4. Machine Learning for Animal Welfare

Machine learning plays a critical role in bridging the gap between raw sensor measurements and the interpretation of welfare-relevant physiological characteristics [17]. While sensing technologies provide continuous data streams reflecting aspects of animal behavior and physiology, these measurements alone do not inherently convey information about the presence, magnitude, or persistence of stressors, nor do they directly indicate an animal’s overall state of welfare.

Through pattern recognition and temporal analysis, ML systems can identify relationships between measurable signals and underlying physiological processes, supporting the detection and characterization of changes in animal state [3,54]. This capability is particularly relevant for distinguishing transient, adaptive responses from sustained conditions with meaningful welfare implications. An example of this would be differentiating a brief metabolic thermoregulatory response from a prolonged environmental heat load.

Critically, ML does not measure welfare directly but operates on proxies derived from sensor data. The validity of these proxies depends on both the quality of the sensing system and the physiological relevance of the features being modeled [71,72]. Without careful alignment between sensor outputs and established physiological mechanisms, models risk detecting statistically consistent patterns that carry no meaningful biological interpretation. This distinction is essential to maintain if sensor-derived outputs are to inform welfare assessment rather than merely correlate with it.

ML can support an analytical pipeline progressing from detection through interpretation to prediction. Detection involves identifying deviations or patterns within sensor data that indicate a change in physiological or behavioral state. Interpretation contextualizes these changes in relation to known biological responses, enabling classification of the nature and potential significance of the underlying condition [24]. Prediction represents a further step, in which temporal patterns are used to anticipate future states or outcomes based on observed trends. Each stage carries distinct data requirements, validation standards, and interpretive constraints that should be explicitly acknowledged in model development and reporting [34].

Accurate interpretation of these stages depends on establishing an appropriate baseline from which meaningful deviations can be detected. Rather than relying on fixed thresholds, welfare-oriented ML systems increasingly employ adaptive baselines that evolve as additional physiological and behavioral data are collected. These baselines may be developed at the individual, herd, or species level depending on the application, although individual-level models generally provide greater sensitivity to subtle physiological changes. Common updating approaches include moving averages, exponential smoothing, and Bayesian estimation, allowing expected ranges to adapt while reducing the influence of transient fluctuations [12]. Despite these advances, establishing reliable baselines for newly deployed systems and highly variable production environments remains an important challenge, emphasizing the need for longitudinal datasets and physiologically informed model development.

The integration of ecological and environmental data streams further enhances the contextual interpretation of physiological measurements [5]. Variables such as ambient temperature, humidity, pasture quality, terrain, resource availability, weather patterns, stocking density, and social interactions influence normal physiological and behavioral responses and may substantially alter the biological significance of a given sensor measurement. Incorporating these complementary data sources within multimodal ML frameworks provides an opportunity to distinguish adaptive responses to changing ecological conditions from sustained physiological deviations that may indicate compromised welfare [73]. Rather than treating environmental measurements as independent variables, future welfare-oriented systems should leverage them as contextual information that improves the biological interpretation of animal-derived sensor data.

#### Ecological Context Within Multimodal Welfare Assessment

Physiological responses do not occur independently of the environment in which an animal lives. Ecological context—including stocking density, social hierarchy, resource availability, housing design, management practices, and environmental conditions—shapes both physiological responses and their interpretation. In extensive systems, pasture quality, terrain, weather, and water availability further influence animal behavior and physiology. These factors also affect what should be considered an individual’s normal state, reinforcing the need for adaptive rather than static baseline models.

Many of these variables can be measured directly using existing PLF technologies (Table 6). Environmental sensors monitor climate conditions, vision systems quantify animal spacing and feeding competition, and positioning systems and accelerometers characterize movement and resource utilization. Machine learning can integrate ecological data with individual physiological and behavioral measurements to improve welfare interpretation. For example, elevated respiration rates observed across multiple animals alongside rising temperature–humidity index may indicate heat stress, whereas similar responses coupled with increased crowding and reduced feeding may instead suggest resource competition. Ecological context therefore serves as an interpretive layer that strengthens the biological relevance of sensor-derived measurements.

### 6.5. Challenges in Adopting Machine Learning in Precision Livestock Farming

A fundamental challenge across all ML applications in livestock systems is generalization. Models trained on controlled datasets frequently fail to perform reliably in commercial environments due to differences in sensor configuration, animal populations, environmental conditions, and management practices [13]. This limitation underscores the dependency of ML performance on the quality and representativeness of the underlying sensing and data acquisition pipeline, reinforcing the need for robust, real-world data collection strategies [14]. A related and often overlooked assumption is that models developed and validated on individual animals will transfer directly to multi-animal environments. This assumption/generalization introduces additional sources of variability and signal ambiguity that single-animal models are not designed to accommodate [17].

Labeling constraints impose additional limitations on the development and deployment of ML models in livestock settings. High-quality labeled datasets require substantial manual effort, domain expertise, and often invasive or inherently subjective ground-truth measurements [3]. In many cases, labels such as “stress” or “health status” cannot be directly quantified and must be inferred, which introduces uncertainty and potential bias into model training [34]. This challenge is particularly consequential when attempting to align ML outputs with meaningful welfare indicators, as relying on simplified proxy labels may lead to models that are statistically functional yet biologically superficial. Addressing this challenge requires larger datasets and careful curation process to accurately represent the physiological and contextual intricacies of the conditions being modeled. Furthermore, physiological labels should account for the dynamic nature of biological regulation, as normal responses vary between individuals and across environmental and production contexts [24]. Developing datasets that incorporate adaptive, physiologically grounded baselines rather than fixed population thresholds will improve both the generalizability of the model and the biological interpretability of ML-derived predictions [31].

Deployment limitations pose an additional barrier to the practical implementation of ML in PLF systems. Constraints on computational resources, connectivity, power availability, and system integration restrict the feasibility of complex models—particularly in edge or resource-limited environments [6]. These constraints create a persistent disconnect between models developed under research conditions and those that can be realistically implemented and maintained in commercial livestock operations. This gap will require coordinated advances in both hardware capability and model efficiency to close.

#### Farmer Adoption and Privacy

The practical success of precision livestock farming technologies depends not only on technical performance but also on producer acceptance, ease of implementation, and long-term operational value [74]. Systems requiring extensive maintenance, specialized technical expertise, or frequent calibration may present barriers to adoption despite demonstrating strong performance under experimental conditions. Likewise, the increasing use of cloud-based analytics and networked sensing platforms has introduced considerations regarding data ownership, privacy, and sharing among producers, technology providers, and researchers [75]. Although these topics extend beyond the scope of sensing technologies, they represent important practical considerations that will influence the widespread adoption and sustained use of welfare-oriented PLF systems.

## 7. From Signal to Inference: A Respiration Sensor Illustrative Translational Workflow in PLF System Design

**Figure 8:**
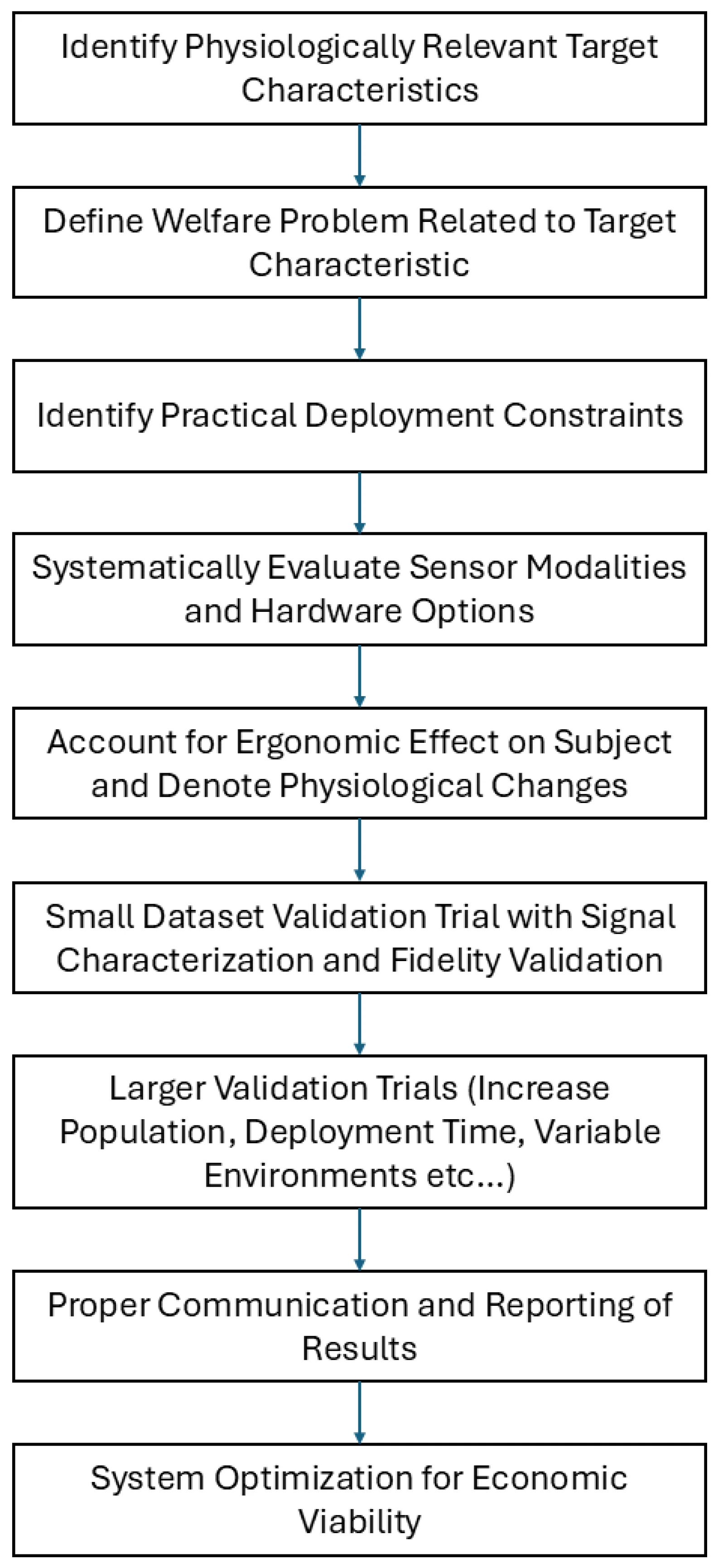
Summary of Translational Workflow/Step-By-Step Guide.

The preceding sections of this review have examined sensing modalities, data acquisition architectures, and machine learning methodologies from a broad, evaluative perspective. To illustrate how these concepts converge during system development, this section presents an example engineering workflow for the design, development, and preliminary validation of a wearable respiration monitoring system for cattle. Figure 8 summarizes the process as a step-by-step workflow, progressing from physiological characteristic selection and deployment constraint identification through sensor selection, hardware development, and signal validation. This example is intended to demonstrate the engineering design process rather than present a statistically validated commercial sensing system. Although illustrated using cattle, the same workflow is broadly applicable to other livestock species, with modifications to sensor selection, ergonomics, validation procedures, and deployment considerations reflecting species-specific anatomy, behavior, and production systems.

### 7.1. Physiological Characteristic and Problem Definition

The development of welfare-focused sensing systems should begin by identifying physiologically relevant target characteristics rather than simply selecting a sensing technology. Physiological characteristics such as respiration rate, body temperature, locomotion, feeding behavior, or posture represent measurable manifestations of an animal’s interaction with its environment and internal physiological state [5]. However, these characteristics rarely correspond to a single biological process or welfare outcome. For example, changes in respiratory dynamics may result from thermoregulatory demand, metabolic activity, physical exertion, disease processes, behavioral responses, or combinations thereof [66]. Due to this, physiological measurements should not be interpreted in isolation but rather within the broader biological and environmental context in which they occur. This distinction is particularly important because sensing systems do not directly measure welfare, stressors, or management outcomes [66]. Instead, they measure physiological or behavioral characteristics that may provide insight into an animal’s response to internal and external stimuli [32]. The value of a sensing system therefore depends on its ability to detect a measurable signal and the extent to which that signal can be reliably linked to a physiologically meaningful characteristic. Establishing this relationship requires careful consideration of both the biological process being monitored and the practical limitations of measurement.

Once the target physiological characteristic has been identified, practical deployment constraints must be considered. Factors such as animal size, behavior, movement patterns, environmental exposure, mounting location, and intended deployment duration frequently impose greater design limitations than the sensing technology itself. In wearable systems specifically, improper mounting can reduce data quality, damage equipment, or alter animal behavior in ways that confound the measurements being collected. Ergonomic design should therefore be treated as a primary engineering constraint, guiding subsequent decisions regarding sensor selection, enclosure design, communication hardware, and power requirements rather than as a secondary consideration addressed after hardware selection.

### 7.2. Sensor Selection and Hardware Development

After physiological objectives and deployment constraints have been established, candidate sensing modalities can be systematically evaluated. Multiple approaches may measure the same physiological characteristic, each presenting distinct trade-offs in sensitivity, specificity, cost, robustness, and deployment practicality. For respiration monitoring, candidate approaches considered included tensile force sensing, accelerometer-based measurements, radar-based detection, and commercial respiration monitoring systems which were retained for comparative validation (Table 7). Sensor selection should ultimately be determined by the ability of a modality to reliably capture the target physiological phenomenon under the specific environmental and operational conditions of intended deployment, rather than by performance benchmarks established under controlled laboratory conditions.

### 7.3. Ergonomics and Welfare Effect of Sensor Placement

Once the physiological characteristic of interest has been identified, the sensing system must be designed around the animal rather than the sensing technology. Animal size, anatomy, movement patterns, housing conditions, social interactions, and expected deployment duration frequently impose greater engineering constraints than the sensing element itself. These considerations influence sensor placement, enclosure geometry, attachment methods, power requirements, communication hardware, and overall system durability.

Wearable sensing systems introduce additional considerations in welfare-oriented applications: the sensing device itself should not become a source of discomfort or behavioral alteration. Components such as collars, harnesses, respiration belts, ear tags, leg-mounted sensors, and implanted devices may influence normal movement, social behavior, or physiological responses if improperly designed or deployed [2,62]. Therefore, ergonomic development should seek to minimize interference with natural animal behavior while maintaining adequate sensor stability and signal quality. The engineering objective is therefore to maximize measurement fidelity while minimizing the physical and behavioral footprint of the sensing system.

The respiration rate monitoring system developed by the authors provides an illustrative example of this design philosophy. Multiple enclosure geometries, belt configurations, mounting locations, and sensing element arrangements were evaluated to balance measurement stability with animal comfort and long-term wearability. This iterative design process demonstrated that seemingly minor ergonomic modifications substantially influenced both signal quality and practical deployment, emphasizing that successful welfare sensing depends as much on thoughtful integration with the animal as on sensor performance alone.

### 7.4. Data Acquisition and Embedded System Design

The selected sensing element must be integrated into a data acquisition system capable of collecting, storing, and transmitting physiological signals. Sampling requirements, power consumption, physical footprint, communication needs, and intended deployment duration govern hardware selection. Microcontrollers, single-board computers, and dedicated embedded acquisition platforms each offer different balances between computational capability and power efficiency, and the appropriate choice depends on the demands of the target sensing modality and deployment environment (Table 8). The selected architecture should be sufficient to capture physiologically relevant signals at the required resolution and sampling rate while minimizing unnecessary system complexity. In practical livestock deployments, robustness, battery longevity, and operational reliability consistently take precedence over raw computational performance. They have the highest priority and should be reflected in hardware decisions from the outset rather than addressed through post hoc design modifications.

### 7.5. Small Dataset Validation and Signal Characterization

Initial validation should focus on determining whether the sensing system is reliably measuring the intended physiological characteristic. Small-scale trials, laboratory testing, and pilot animal deployments provide opportunities to compare sensor outputs against reference measurements while characterizing signal quality, noise sources, and measurement consistency. Signal processing techniques, including filtering and Fast Fourier Transform (FFT) analysis, can be used to isolate physiologically relevant frequency components from motion artifacts and environmental noise like Figure 7. At this stage, the objective is not prediction or welfare assessment, but verification of the sensing mechanism itself, serving as confirmation that the system captures the target signal with sufficient fidelity to support subsequent analytical development.

Following initial validation, sensing systems must be evaluated across larger animal populations, longer deployment durations, and more variable operational environments. Increased scale introduces challenges associated with sensor drift, environmental variability, inter-animal differences, communication reliability, and data volume management. Critically, successful scaling is contingent on preserving the signal quality and physiological relevance established during initial validation. The scale of the experiment alone does not improve a poorly characterized signal, and unresolved measurement artifacts will propagate and compound across larger datasets. When signal integrity is maintained, expanded datasets provide the foundation for anomaly detection, temporal analysis, machine learning model development, and multimodal integration.

The example preliminary trials demonstrated that the prototype generated repeatable respiratory waveforms suitable for FFT-based signal processing. However, the limited number of animals and exploratory nature of the study did not produce conditions for statistically robust evaluation of measurement accuracy or generalization. Because of this, the subsequent stages of the workflow focus on larger-scale validation, multimodal confirmation, and machine learning development, where statistically meaningful datasets become available.

### 7.6. Interpretation, Reporting, and Scientific Communication of Results

Conclusions drawn from PLF sensing systems should clearly reflect what was directly measured, what was inferred, and what remains hypothetical. In the respiration sensing example presented here, the direct measurement was a periodic change in sensor output corresponding to circumferential displacement of the thoracic region during breathing. Signal processing identified a dominant frequency consistent with respiratory motion, supporting the interpretation that the measured signal was associated with respiratory activity.

The four respiration sensors successfully detected thoracic motion associated with respiratory activity across all four sensing approaches evaluated (Table 9) [66]. In a clinical trial, the radar sensor readings matched well with those from a clinical standard vital monitoring equipment based on visual inspections. This demonstrated that the radar sensor has potential on stable, non-contact measurements without requiring direct attachment to the animal. The tensile force and accelerometer-based sensors required more extensive post-acquisition signal processing and produced reliable respiration measurements when appropriate sensor attachment was achieved, making both strong candidates for cost-effective wearable deployment. The commercial respiration sensor yielded accurate measurements; however, the requirement for proprietary software increases total system cost substantially, limiting its practicality for large-scale or field-based PLF applications.

Nonetheless, additional validation is required before concluding that any of these signals exclusively represents respiration, or that the derived respiratory measure reflects a particular welfare state. These interpretive boundaries apply broadly across PLF systems, where sensors characteristically measure physiological or behavioral proxies rather than welfare directly. Maintaining explicit distinctions between observation, interpretation, and inference is essential for scientific rigor and for the development of sensing technologies that are both biologically meaningful and operationally trustworthy.

### 7.7. Economic Considerations

Economic feasibility remains one of the primary determinants of whether precision livestock technologies transition from research environments to widespread commercial adoption. However, cost should not be viewed solely as a barrier to implementation. Rather, it represents a design/functionality constraint [76].

The cost of PLF systems spans several orders of magnitude, ranging from inexpensive embedded microcontrollers and environmental sensors costing only a few dollars to advanced thermal imaging systems, radar platforms, or multi-sensor networks costing thousands of dollars per installation (Table 10). Likewise, hardware acquisition represents only one component of the total investment. Infrastructure, power systems, communication networks, installation, maintenance, calibration, software licensing, and labor frequently contribute substantially to the overall lifecycle cost of deployment [67].

In contrast, continued reductions in the cost of embedded electronics, wireless communication, and computational hardware have substantially lowered the barrier to entry for many sensing applications. Affordable microcontrollers, single-board computers, and open-source software ecosystems increasingly enable the development of customized sensing platforms that were previously economically impractical. Hence, economic considerations should emphasize value rather than cost alone, balancing measurement capability, operational reliability, and expected management benefit within the context of the intended production system.

## 8. Discussion and Future Directions

This review examined the current state of precision livestock farming (PLF) technologies, with particular focus on sensing modalities, data acquisition (DAQ) systems, and machine learning (ML) methodologies as they relate to welfare-relevant physiological characteristics. Sensing domains including infrared thermography, radar-based sensing, vision systems, acoustic monitoring, and wearable technologies have each advanced in their capacity to continuously capture physiological and behavioral data at the individual animal level. In parallel, advances in DAQ infrastructure and analytical frameworks have expanded the ability to collect, process, and transmit increasingly large and complex datasets in near real time. Collectively, these developments demonstrate the growing feasibility of integrating automated sensing systems into commercial livestock production environments.

Despite these advances, a persistent disconnect remains between the demonstrated capabilities of proposed PLF systems and their reliable implementation in commercial livestock environments. Most of the current literature evaluates sensing systems under highly controlled or semi-structured conditions that reduce measurement variability in ways that do not reflect the realities of production settings. Sensors are frequently validated under conditions in which animals are restrained, isolated, or deliberately positioned to optimize signal quality. These practices artificially eliminate challenges such as motion artifacts, occlusion, environmental noise, variable lighting, dust exposure, and sensor placement instability. While controlled evaluations are valuable for establishing conceptual viability, they do not adequately address the practical barriers to real-world deployment, including scalability, long-term maintenance, system integration, and the economic feasibility of adoption at a commercial scale.

This discrepancy becomes increasingly consequential when evaluating the scalability and reliability of sensing systems intended for welfare assessment. Environmental variability, infrastructure limitations, power constraints, communication robustness, maintenance requirements, and cost–benefit feasibility collectively constrain real-world deployment in ways that controlled studies do not capture. Systems demonstrating high accuracy under optimized experimental conditions frequently experience significant performance degradation when subjected to the dynamic and unpredictable conditions of commercial livestock environments. Greater emphasis must therefore be placed on robustness, repeatability, maintainability, and long-term operational performance under realistic deployment conditions, as isolated performance metrics from controlled settings offer limited insight into a system’s true utility for welfare-oriented applications.

Another major finding in this review is the significant ambiguity in interpreting the physiological and behavioral measurements within the broader context of animal welfare. Many studies associate a wide range of measurable physiological responses with “stress” while failing to adequately distinguish between transient adaptive responses and sustained physiological states that may meaningfully impact welfare. Stressors are not inherently detrimental and may represent normal biological adaptation, arousal, environmental responsiveness, or acute compensatory regulation. The welfare relevance of any given response is determined not by the response itself but by its magnitude, duration, persistence, and the physiological context in which it occurs.

Consequently, the interpretation of welfare-relevant sensing data requires substantially more precise physiological baseline than is currently reflected in the literature, where context is frequently absent, inconsistently applied, or insufficiently justified. This review emphasizes that sensing technologies fundamentally measure physiological or behavioral outputs rather than welfare itself, and that the biological meaning assigned to these measurements must be approached with deliberate intentionality. Without this foundation, associations between sensor-derived data and welfare states risk remaining correlational rather than mechanistically grounded, limiting their scientific validity and their utility for evidence-based welfare management.

Recently, machine learning (ML) has emerged not as an independent solution but as a critical analytical bridge between raw sensor measurements and meaningful interpretation of physiological patterns. ML frameworks can identify relationships, patterns, and temporal dynamics within large, multimodal datasets that would otherwise be difficult to resolve using conventional analytical methods. Through supervised learning, deep learning, time-series analysis, and multimodal integration, ML systems may support progression from signal detection to contextual interpretation and, ultimately, prediction of welfare-relevant physiological states. However, the effectiveness of these systems remains fundamentally dependent on the quality, consistency, and physiological relevance of the data used for training and validation. Poorly collected datasets, inadequate labeling methodologies, environmental variability, and insufficient model generalization across production environments represent persistent and interrelated barriers to the practical application of ML within PLF systems. The central argument of this review is that future advancement of welfare-oriented PLF systems depends less on increasing the complexity of sensing hardware or ML models and more on strengthening the relationships among sensing methodologies, physiological context, and real-world deployment constraints. Future systems must move beyond demonstrations of technical capability under idealized conditions and instead prioritize physiologically meaningful, operationally robust sensing pipelines that function reliably in commercial livestock environments. Realizing this requires the development of standardized validation methodologies, longitudinal multimodal datasets collected under commercial conditions, and physiology-based frameworks for interpreting sensor-derived data within appropriate biological context. Achieving this vision will require sustained interdisciplinary collaboration among engineers, computer scientists, physiologists, animal behaviorists, and livestock producers—ensuring that future PLF systems remain technologically capable, biologically grounded, and practically deployable.

Beyond technological capability, successful implementation of precision livestock farming systems ultimately depends on practical adoption by producers and industry stakeholders. Sensing systems must be economically justifiable, simple to install and maintain, and sufficiently robust to withstand demanding commercial environments characterized by dust, moisture, vibration, and continuous animal interaction. Routine calibration requirements, battery replacement, hardware failures, and system maintenance all contribute to the long-term operational cost of deployment and may limit adoption despite promising experimental performance.

User acceptance represents an equally important consideration. Producers must be able to interpret system outputs with confidence and understand how sensor-derived information supports management decisions without replacing professional judgment. Transparent analytical frameworks and physiologically interpretable outputs are therefore likely to promote greater trust than highly complex models that function as “black boxes.” As commercial PLF systems continue to expand, additional considerations of data ownership, privacy, and interoperability among manufacturers will also become increasingly important, particularly as large volumes of animal and farm management data are integrated across multiple platforms.

A critical evaluation of the perspective and potential limitations of this review is warranted. The strong emphasis on practical deployment constraints and physiological specificity may bias discussion toward conservative assessments of current technological capabilities, potentially undervaluing emerging technologies that have not yet achieved large-scale deployment but hold meaningful long-term promises. Similarly, the deliberate effort to distinguish welfare-relevant physiological characteristics from generalized notions of “stress” and production-related outcomes reflects a particular conceptual framing that is not universally adopted across animal science or PLF research. While this distinction is intended to improve the interpretive rigor of physiology-based sensing data, it may underrepresent broader behavioral, environmental, and management indicators that are meaningful to welfare assessment in applied settings. 

Future research should prioritize developing scalable sensing architectures that balance data fidelity with practical deployment requirements. Hybrid edge–cloud processing systems, adaptive sampling strategies, low-power communication protocols, and multimodal sensor fusion represent promising directions for improving operational scalability while preserving analytical utility. Reducing dependence on extensively curated labeled datasets is an equally important priority, particularly in applications where physiological states are difficult to define directly or where subjective interpretation introduces systematic uncertainty into model training. Progress in self-supervised, semi-supervised, and transfer learning approaches may offer viable pathways toward more generalizable models that perform reliably across the heterogeneous conditions of commercial livestock environments. 

## 9. Conclusions

The rapid growth of precision livestock farming (PLF) reflects increased integration of sensing technologies, data acquisition systems, and artificial intelligence within animal production systems [77]. Despite substantial technological advancement, a consistent theme throughout the literature is the gap between successful experimental demonstrations and reliable deployment under commercial livestock conditions. Environmental variability, unrestricted animal movement, infrastructure limitations, sensor durability, and biological complexity continue to challenge the translation of many promising technologies into practical welfare-monitoring tools.

Across the reviewed sensing modalities, no single technology provides a complete solution for welfare assessment. Infrared thermography, radar, vision-based systems, acoustic monitoring, and wearable sensors each offer unique strengths while simultaneously introducing limitations related to measurement accuracy, deployment feasibility, scalability, or physiological interpretation. As a result, the future of welfare-oriented PLF systems will likely depend less on identifying a singular sensing technology and more on effectively integrating complementary sensing modalities with robust data acquisition and analytical frameworks.

Machine learning (ML) provides an important bridge between raw sensor measurements and meaningful physiological interpretation. However, the performance of ML systems remains fundamentally dependent upon the quality, representativeness, and physiological relevance of the underlying data. Advances in algorithm development alone cannot compensate for limitations in sensing methodologies, inadequate validation, or poorly defined biological relationships. Future research should therefore prioritize large-scale field validation, multimodal sensing frameworks, improved generalizability across production environments, and stronger connections between measured characteristics and underlying physiological mechanisms.

Ultimately, the objective of welfare-oriented PLF should not be to directly measure welfare itself, but rather to generate physiologically meaningful, operationally reliable, and contextually interpretable information that supports welfare assessment. Continued progress will require critical evaluation of what is truly being measured, what can reasonably be inferred from those measurements, and how those interpretations translate across diverse commercial production systems. By aligning sensing capabilities with physiological understanding and practical deployment realities, future PLF systems may move beyond technological demonstration toward meaningful contributions to animal welfare monitoring and management.

## Figures and Tables

**Table 1:** Classification of Stressors with Respect to Physiological Characteristics, Welfare Relevance, and Relation to Ability to be Sensed.

Stressor Classification	Magnitude	Duration	Physiological Characterization	Welfare Relevance	Recovery Potential	Typical Sensor-Relevant Characteristics
Mild/Transient	Low	Short	Temporary adaptive physiological activation without substantial disruption of basal function	Low unless cumulative or frequent	High	Minor thermal fluctuation, brief activity changes, transient respiration elevation
Acute High-Intensity	High	Short	Rapid physiological activation with temporary diversion of metabolic and regulatory resources	Moderate to high depending on severity and recovery	Moderate to high if recovery occurs rapidly	Elevated respiration, abrupt movement, thermal redistribution, vocalization changes
Repeated Acute	Moderate	Intermittent	Recurrent activation events with incomplete physiological recovery between exposures	Increasing welfare relevance due to cumulative physiological demand	Variable depending on frequency and recovery interval	Repeated activity alteration, prolonged elevated respiration, altered behavioral patterns
Chronic Low-Level	Low–Moderate	Long	Sustained low-level physiological burden with gradual resource depletion and adaptive compensation	Moderate due to prolonged persistence and cumulative effects	Reduced over time	Persistent behavioral modification, altered feeding/activity trends, long-term thermal shifts
Chronic High-Level	High	Long	Prolonged disruption of physiological regulation with impaired recovery and elevated metabolic demand	High due to sustained physiological compromise	Low without intervention	Sustained thermal dysregulation, chronic locomotion alteration, persistent abnormal respiration or behavior

**Table 2:** Comprehensive Overview of Sensor Modalities.

Modality	Sensor Type	Measured Variable	Desired Measurement Extrapolation	Deployment Mode	Strengths	Limitations	References
Infrared/Thermal	Infrared Thermography (IRT) Camera	Surface Temp	Change/difference in temperature	Fixed, Non-Contact	Non-invasive, multi-animal monitoring, thermal mapping	Environmental sensitivity, occlusion, reduced precision at distance	[35]
	Point Infrared Temperature Sensor	Local Temp	Change/difference in temperature	Fixed, Non-Contact	High localized precision, simple integration	Narrow detection area, requires alignment and close range	[36]
Radar	Frequency-Modulated Continuous Wave (FMCW) Radar	Respiration	Respiration rate	Fixed, Non-Contact	Non-contact vital monitoring, low light capability	Motion interference, signal complexity	[37]
	Doppler	Heart/Resp	Heart and respiration rate	Fixed, Non-Contact	Fine motion detection, physiological monitoring	Limited range resolution, noise sensitivity	[37]
	Ultra-Wideband (UWB) Radar	Position + vitals	Movement characteristics	Fixed, Non-Contact	Penetrates dust/occlusion, spatial tracking	High processing demand, regulatory complexity	[37,38]
Vision	Red-Green-Blue (RGB) Camera	Behavior	Movement characteristics	Fixed, Non-Contact	Rich behavioral data, scalable imaging	Lighting dependence, occlusion, tracking difficulty	[39,40,41]
	Depth	3D posture	Movement characteristics	Fixed, Non-Contact	3D spatial analysis, posture estimation	Limited range, environmental interference	[39,40,41]
	Thermal cam	Temp + image	Movement and temperature characteristics	Fixed, Non-Contact	Combines thermal and behavioral analysis	High cost, calibration complexity	[39,40,41]
Acoustic	Microphones	Sound	Respiration rate	Fixed Contact or Non-Contact	Passive monitoring, scalable deployment	Background noise, individual identification difficulty	[14]
Wearable	Inertial Measurement Unit (IMU)	Movement	Movement characteristics	Fixed, Contact	High temporal resolution, robust activity tracking	Attachment stability, indirect physiology	[8]
	Global Positioning System (GPS)	Position	Location and movement	Fixed, Contact	Large-area tracking, grazing behavior analysis	Battery demand, poor indoor accuracy	[7]
	Radio-Frequency Identification (RFID)	Events	Location and movement	Fixed, Contact and Non-Contact	Reliable identification, low cost	Sparse/event-only data, infrastructure dependent	[42]
	Rumen bolus	Temp/pH	Rumen characteristics	Internal	Direct internal physiological measurement	Cost, lifespan, limited behavioral context	[29]

**Table 3:** Summary of Sensor Study Limitations and Shortcomings.

Source of Uncertainty	Limiting Factor	Potential Outcome/Interpretation
Small sample size	Limited number of animals or experimental replicates	Reduced statistical power; findings may not generalize across populations or production systems.
Single species or breed	Homogeneous study population	Results may not transfer to other breeds, species, ages, or production systems.
Controlled experimental environment	Stable lighting, climate, housing, and management	Performance may degrade under commercial conditions with greater environmental variability.
Short study duration	Limited observation period	Unable to assess long-term performance, adaptation, seasonal effects, or chronic welfare changes.
Artificially induced conditions	Experimentally generated stressors or behaviors	Responses may differ from naturally occurring physiological or behavioral events.
Limited ground-truth validation	Reliance on proxy measurements or subjective observations	Weaken confidence that measured signals accurately represent the intended physiological characteristic.
Single sensing modality	Dependence on one sensor type	Important contextual information may be omitted, increasing ambiguity in interpretation.
Limited environmental variability	Few changes in weather, housing, or management	Model robustness and sensor performance under diverse conditions remain unknown.
Sensor placement variability	Differences in mounting location, orientation, or animal fit	Increased measurement variability and reduced repeatability between studies.
Sparse or subjective labels	Labor-intensive annotation or indirect welfare labels	Machine learning models may learn inaccurate or biologically weak relationships.
Limited scalability	Small experimental systems	Unknown performance in large commercial herds or flocks.
Animal–device interaction	Wearable discomfort, altered behavior, or equipment damage	Sensor may influence the physiological or behavioral characteristic being measured.
Human management effects	Differences in handling, feeding, or routine management	Confounding factors may influence measured responses independently of welfare status.

**Table 4:** Overview of Data Acquisition Hardware for Precision Livestock Farming.

Category	Modality	Description	Typical Technologies	Data Characteristics	Advantage	Limitation	Deployment Context
Embedded Acquisition	Microcontrollers	Low-power embedded systems for sensor interfacing and signal acquisition	Arduino, ESP32, STM32	Low-to-moderate throughput, real-time sampling	Low cost, energy efficient, compact	Limited processing power and memory	Wearable systems, distributed sensor nodes
Embedded Acquisition	Microprocessors	Higher capability embedded processing systems	ARM processors, embedded Linux systems	Moderate-to-high throughput	Supports multitasking and advanced preprocessing	Higher power consumption	Fixed sensing stations, edge systems
Embedded Acquisition	Single-Board Computers (SBCs)	Compact integrated computing platforms for acquisition and local processing	Raspberry Pi, NVIDIA Jetson, BeagleBone	High data throughput, multimodal support	Supports ML inference and image processing	Environmental durability and thermal constraints	Vision systems, edge AI deployments
Embedded Acquisition	Dataloggers	Standalone acquisition and storage systems	Campbell Scientific, HOBO loggers	Buffered local storage	Reliable long-term recording	Limited real-time capability	Environmental and physiological monitoring
Embedded Acquisition	PLC Systems	Industrial-grade control and acquisition systems	Siemens PLC, Allen-Bradley	Deterministic and stable acquisition	High reliability and industrial robustness	High cost and reduced flexibility	Automated barns, industrial feeding systems
Embedded Acquisition	Edge AI Devices	Localized acquisition and AI-enabled preprocessing hardware	NVIDIA Jetson, Coral TPU	Real-time multimodal processing	Reduced cloud dependency and latency	Power and cooling requirements	Advanced smart farm systems

**Table 5:** Overview of Data Communications for Precision Livestock Farming.

Category	Modality	Description	Typical Technologies	Data Characteristics	Advantages	Limitations	Deployment Context
Infrastructure	Wired Systems	Fixed physical connections for data transmission	Ethernet, serial communication, fiber optics	High bandwidth, low latency, continuous	Highly reliable and stable	High installation cost and low flexibility	Milking parlors, feeding stations
Infrastructure	Wireless Systems (WSN)	Distributed wireless sensor communication networks	Zigbee, Wi-Fi, BLE, mesh networks	Variable bandwidth, intermittent transmission	Flexible and scalable	Signal interference and power constraints	Barns, wearable systems
Communication Range	Short-Range Communication	Localized communication within confined areas	BLE, Zigbee, Wi-Fi	High data rate, low latency	Suitable for high-frequency sensing	Limited range	Indoor barns and feeding areas
Communication Range	Long-Range Communication	Remote communication across extensive areas	LoRaWAN, cellular, satellite	Low bandwidth, long-distance transmission	Large coverage area	Latency and limited throughput	Pasture and grazing systems
Architecture	Edge Computing	Localized preprocessing near acquisition source	Embedded processors, SBCs	Reduced transmission burden	Low latency and real-time capability	Limited computational scalability	Remote deployments
Architecture	Cloud Computing	Centralized storage and analytical infrastructure	Cloud servers and remote platforms	Large aggregated datasets	High computational capability	Connectivity dependence	Centralized farm systems
Architecture	Hybrid (Edge + Cloud)	Combined local and centralized processing	Edge devices with cloud integration	Balanced data flow	Improved scalability and latency balance	Increased system complexity	Integrated PLF systems
Sampling Strategy	High-Frequency Sampling	Continuous rapid acquisition	IMUs, thermal sensors, acoustic systems	High temporal resolution	Captures higher temporal resolution for transient event detection	High storage and power demand	Intensive monitoring applications
Sampling Strategy	Low-Frequency Sampling	Periodic acquisition at relatively long sampling intervals.	RFID, GPS, environmental sensors, slow physiological sensors	Sparse, low temporal resolution	Low power consumption, reduced storage and communication requirements, extended deployment duration	May miss transient physiological or behavioral events; reduced temporal fidelity	Long-term monitoring, pasture systems, battery-powered deployments
Sampling Strategy	Event Detection Based Sampling	Only when predefined thresholds or events are detected.	Motion-triggered cameras, threshold-based accelerometers, acoustic event detection	Event-driven, intermittent	Efficient data collection, minimizes storage and power usage while capturing relevant events	Dependent on threshold selection; may miss subtle or unexpected events	Behavioral monitoring, anomaly detection, edge-based sensing systems
Sampling Strategy	Adaptive Sampling	Sampling frequency dynamically adjusted according to physiological state, environmental conditions, or detected system changes.	Intelligent microcontrollers, edge AI, embedded adaptive DAQ systems	Variable temporal resolution	Balances signal fidelity with power consumption and computational efficiency; prioritizes periods of interest	Increased algorithmic complexity; requires reliable decision logic and validation	Intelligent embedded sensing platforms, long-term autonomous welfare monitoring

**Table 6:** Levels of Multimodal Data Streams within PLF Systems.

Data Source	Representative Measurements	Primary Information Obtained	Role in Welfare Interpretation
Individual Animal	Respiration rate, heart rate, body temperature, posture, locomotion	Physiological regulation and individual behavioral responses	Detect changes occurring within an individual animal
Social/Group-Level	Feeding synchronization, crowding, social interactions, movement distribution, resource utilization	Social behavior and collective adaptive responses	Provides context for distinguishing individual abnormalities from population-level events
Environmental	Ambient temperature, humidity, THI, airflow, light intensity, noise, pasture conditions	External stressors and production environment	Identifies environmental drivers influencing physiological and behavioral responses
Integrated Multimodal Model	Combined physiological, behavioral, and environmental data	Context-dependent welfare assessment	Improves interpretation by relating physiological responses to ecological and management conditions

**Table 7:** Respiration Sensor Selection [66].

Sensor Type	Contact Type	Photo	Special Considerations
Radar Sensor	Non-Contact Sensor	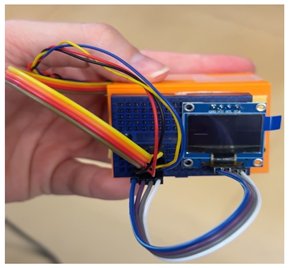	Proximity to Animal and Detection Area
Accelerometer Sensor	Contact Sensor	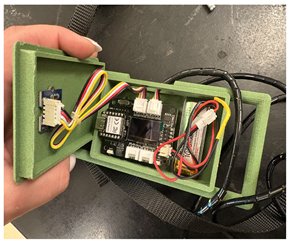	Ergonomics and Placement Positioning
Tensile Force Sensor	Contact Sensor	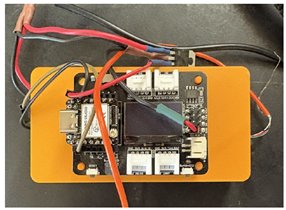	Ergonomics and Placement Tension
Commercial Respiration Sensor	Contact Sensor	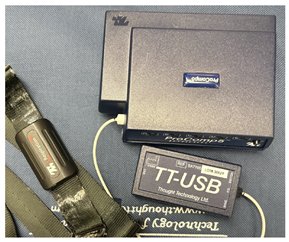	Ergonomics and Placement Tension

**Table 8:** Data Acquisition for the Respiration Sensors [66].

Type	Data Acquisition	Ability	Footprint
Arduino Based	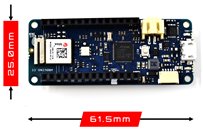	Low Power Solution with SD card compatibility and moderate resolution analog to digital conversion ability	Medium
ESP32 Based	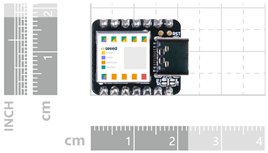	Low Power Solution with SD card compatibility, Wi-Fi, and high-resolution analog to digital conversion ability	Smallest
Raspberry Pi (Single Board Computer)	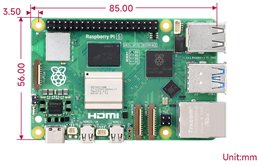	Moderate Power solution with full computer capabilities and high-resolution analog to digital conversion ability	Largest

**Table 9:** Respiration Sensor Performance Comparison [66].

Sensor Type	Contact Type	Performance Summary	Design Weak Points
Radar Sensor	Non-Contact Sensor	Radar sensors work exceptionally well in environments with little occlusion and close relative proximity to the animal. Their resolutions are particularly impressive in said environments. These sensors work exceptionally well on anesthetized animals.	Motion artifacts and signal noise in real-world environments
Accelerometer Sensor	Contact Sensor	Accelerometer-based sensors can provide meaningful positional and movement data with small and simple sensors. With proper fixation upon an animal, transient motion data can be readily collected.	The placement of the sensor and necessity for multiple accelerometers to cross reference one another greatly limits ergonomics. Sensors are often bashed and thrown off animals.
Tensile Force Sensor	Contact Sensor	Tensile force sensors are the standard for human-based respiration sensors. They are simple, effective, and easy to integrate with near any data acquisition system or microcontroller interface. With proper tensioning, accurate respiration waveform data can be produced.	Ergonomics, placement, and tensioning greatly impact the ability of the animal to tolerate such an apparatus. To finely tune tension, the animal must endure discomfort for the duration of fitting and wearing time. Sensors are often bashed and thrown off animals.
Commercial Respiration Sensor	Contact Sensor	Since tensile force sensors are the standard for human-based respiration sensors, a human analog was used for comparison. Human trials showed its effectiveness in a seated and relaxed human. In theory, a similarly immobile and relaxed animal could produce accurate results.	All ergonomics and placement issues are identical to that of the handmade tensile force sensors. However, the addition of a large data logger that must be physically mounted in tandem with the sensor itself made it, so the sensor was impossible to comfortably mount it on an animal.

**Table 10:** Common System Costs At-a-Glance.

System Complexity	Typical Hardware Components	Approximate Cost (USD)	Typical Deployment Scale	Primary Cost Drivers	Primary Adoption Barrier
Educational/Prototype	Microcontroller (Arduino, ESP32), single sensor, basic power supply	$100–$500	Laboratory, proof-of-concept	Individual sensors, prototyping materials	Technical validation
Research Prototype	Multiple sensors, SBC (Raspberry Pi), wireless communication, custom enclosure	$500–1000	Individual animal research	Sensor integration, enclosure fabrication, DAQ hardware	System integration
Advanced Research Platform	Multiple synchronized sensors, embedded computing, edge processing, custom electronics	$1500–4000	Small research herds	Sensor fusion, embedded computing, custom hardware	Computational resources
Commercial (Light Deployment)	Wearables, environmental sensors, gateways, cloud connectivity, commercial software	$5000–50,000 (or ~$200–500 per monitored animal depending on system)	Small to medium farms; targeted monitoring	Proprietary hardware, subscriptions, installation	Initial investment and recurring subscription costs
Commercial (Comprehensive PLF)	Integrated vision, thermal imaging, environmental sensing, wearables, edge/cloud computing, automated analytics	$50,000–500,000+	Large commercial operations and integrated facilities	Multi-sensor infrastructure, networking, software licensing, installation, maintenance	Capital investment, infrastructure, long-term support

## Data Availability

The data presented in this study are available from the corresponding author upon reasonable request.
